# Region-resolved proteomic map of the human brain: functional interconnections and neurological implications

**DOI:** 10.1038/s41392-025-02554-8

**Published:** 2026-02-04

**Authors:** Pei-Pei Zhang, Man-Sheng Li, Jia Zhou, Chu-Hong Zhu, Rui Tang, Zhi-Cheng He, Xiao-Hong Yao, Yi-Fang Ping, Dong-Fang Xiang, Le-Yong Tan, Yu-Jie Wang, Shuai Wang, Si-Si Li, Jie Ma, Yun-Ping Zhu, Xiu-Wu Bian, Ling Leng

**Affiliations:** 1 Chongqing Institute of Advanced Pathology and Yu-Yue Scientific Research Center for Pathology, Jinfeng Laboratory, Chongqing, China; 2 Institute of Pathology, Medical Research Center for Glioma, Southwest Cancer Center, the First Affliated Hospital (Southwest Hospital) and School of Basic Medical Sciences, Army Medical University (Third Military Medical University), and the Key Laboratory of Tumor Immunopathology, the Ministry of Education (Third Military Medical University), Chongqing, China; 3 Department of Pathology, Ruijin Hospital and College of Basic Medical Sciences, Shanghai Jiao Tong University School of Medicine, Shanghai, China; 4 State Key Laboratory of Proteomics, National Center for Protein Sciences (Beijing), Beijing, China; 5 Stem Cell and Regenerative Medicine Lab, National Infrastructures for Translational Medicine and State Key Laboratory for Complex, Severe, and Rare Diseases, Institute of Clinical Medicine, Peking Union Medical College Hospital, Chinese Academy of Medical Sciences and Peking Union Medical College, Beijing, China; 6 Department of Anatomy, Third Military Medical University (Army Medical University), Chongqing, China; 7 Jinfeng Laboratory, Chongqing, China

**Keywords:** Molecular neuroscience, Tissue engineering, Gene expression analysis, Genome informatics

## Abstract

While progress has been made in transcriptomic profiling of the human brain, functional characterization of brain regions and their interactions on the basis of regional protein expression remains limited. Here, we constructed a proteomic map from thirteen anatomical brain regions of eight cadaver donors to elucidate region-specific protein expression patterns and their implications for brain function. The results underscore the interconnectivity of the four cerebral lobes, suggesting facilitated information integration through large-scale neural networks. We propose a three-module framework (cortical integration module [frontal lobe, temporal lobe, parietal lobe, occipital lobe], limbic-relay network [amygdaloid nucleus, hippocampus, thalamus/hypothalamus], and midline regulatory axis [thalamus/hypothalamus, corpus callosum, ventricles, optic chiasm]) and provide molecular evidence supporting the potential involvement of the midline regulatory axis, brainstem, and cerebellum in higher-order cognitive functions. The midline regulatory axis may play a critical but underexplored role in neurodevelopment, interregional signaling, and structural homeostasis, potentially through efficient synaptic function, energy metabolism, and extracellular matrix integrity. This analysis may enhance the understanding of brain physiology and highlight the need to integrate proteomic and transcriptomic approaches in the study of brain function and neurological disorders.

## Introduction

The brain orchestrates complex behaviors such as cognition, sensation, and action through coordinated interactions among specialized regions. Humans possess approximately 86 billion cortical neurons and a similar number of glial cells, which are positively correlated with cognitive capacity.^1,2^ Beyond quantitative differences in neuronal and glial populations, the functional specialization of brain regions arises from their distinct molecular architectures, including region-specific neurotransmitter systems, synaptic protein repertoires, and metabolic signatures. These molecular features shape the computational functions of major structures such as the cerebral cortex, cerebellum (CB), and limbic system. The cortex comprises several lobes with well-defined roles: the frontal lobe (FL) governs executive control and motor planning; the parietal lobe (PL) integrates somatosensory and spatial information; the temporal lobe (TL) supports auditory processing and memory; and the occipital lobe (OL) mediates visual perception. Subcortical regions also contribute critically: the amygdaloid nucleus (AN) regulates emotion and salience, the hippocampus (HIP) enables memory formation and navigation, and the thalamus and hypothalamus (THA/HT) coordinate sensory relay, endocrine signaling, and homeostasis. The olfactory bulb and tract (OB/OT), located at the anterior base of the FL, serve as the primary relay for olfactory input. White matter structures such as the corpus callosum (CC) mediate interhemispheric communication, while the ventricles (VT) form cerebrospinal fluid-filled cavities essential for metabolic exchange. The optic chiasm (OC), where retinal fibers partially decussate, is a key node for visual-field processing. The brainstem (BS), comprising midbrain, pons, and medulla, controls vital autonomic and motor functions and links the spinal cord to higher-order centers. Together, these spatially distributed regions exhibit distinct cellular and molecular profiles that underlie the complexity of human brain organization.

Recent high-throughput technologies have enabled the characterization of DNA, RNA, and proteins in the brain, facilitating the investigation of disease networks and potential therapeutic targets.^2^ Studies have explored the molecular mechanisms related to neurological disorders such as Alzheimer’s disease (AD) by constructing gene expression networks to identify key genes.^3^ Large-scale transcriptomic atlases, such as those from the Allen Brain Atlas and GTEx, have further revealed substantial variation in gene expression across brain regions, underscoring the need to integrate multi-omics layers to understand regional vulnerabilities to disease.^4,5^ However, the correlation between RNA and protein expression is weak, as gene expression does not always predict protein abundance due to differing mRNA and protein half-lives, particularly in the complex brain.^6,7^ Additionally, protein abundance is profoundly shaped by cell-type composition, neuronal activity, post-translational modifications, and spatial microenvironment factors that independently vary across brain regions and are not captured at the transcript level.

Proteins regulate various biological functions and can serve as prognostic markers for neurological diseases, including brain cancers.^8^ Proteomic methods are increasingly used to study protein interactions in different cell types, revealing their roles in neuroinflammation and AD.^9^ Spatially resolved proteomics has also begun uncovering brain-region-specific interaction networks, metabolic pathways, and structural protein modules, highlighting the diversity of proteomic landscapes across the human brain.^10^ Integrative approaches combining epigenomic, transcriptomic, proteomic, and metabolomic data have established multilayered biological classifications to elucidate disease mechanisms.^11^ Yet, despite these advancements, systematic regional proteomic mapping of the human brain, including both cortical and subcortical structures and based on multiple individuals, remains insufficient.

Despite extensive comparative proteomic studies in animal models, these studies do not fully capture the changes in protein expression in the human brain under pathological conditions.^12^ For example, existing animal models for AD do not fully recapitulate symptoms.^13^ Species-specific differences in neuronal subtype composition, synaptic density, and cortical expansion profoundly limit the translational relevance of rodent proteomic datasets. Thus, human-based proteomic data are essential for accurately understanding disease mechanisms, regional vulnerability, and molecular pathways underlying neurological disorders. The heterogeneity and complexity of the brain necessitate the use of multiple omics methods to establish interconnections among brain regions. Recent research has integrated transcriptomics and proteomics to reveal the multilayered structure of gliomas.^14^ However, comprehensive proteomic analyses across entire brain regions are limited.

Currently, many neuroscience studies have focused on the relationships between different anatomical structures of the human brain and behavior. Because of their diverse cell types and functions, protein expression levels are different in various regions of the brain.^15^ A recent study identified 123 reference proteins in three mouse brain regions via proteomics, along with eight alternative proteins linked to brain functions.^10^ Another study presented a quantitative protein atlas with over 11,000 proteins across 13 human brain regions in one human brain, revealing distinct protein expression patterns and functional differences.^16^ However, the reliance on a single donor limits generalizability, and broader regional coverage across multiple human brains is needed to robustly define conserved protein signatures.

Compared with these studies, our research presents a more comprehensive approach. Unlike studies utilizing animal models, this work is based on human brain samples, allowing for a more accurate reflection of biological processes and pathological features within the human nervous system. The employment of eight human brain samples enhances the statistical power and representativeness. Covering 13 brain regions that encompass the entire brain provides a broader perspective. This study explores functional interconnections among different brain areas through regional resolution, laying the groundwork for further explaining various disease models.

## Results

### Region-resolved proteome architecture and three-module framework

**Fig. 1:**
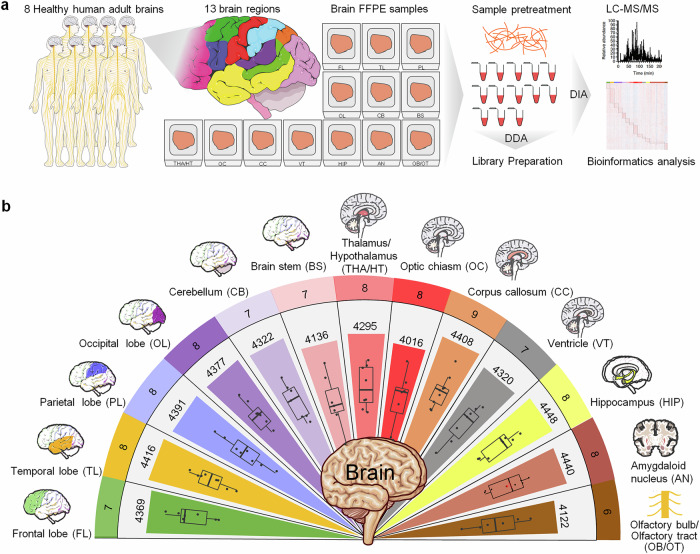
Quantitative proteome profiling of regionally distinct protein signatures in human brain tissues. **a** Schematic diagram of the experimental workflow. This includes sample collection, acquisition of quantitative proteomes, and bioinformatics analysis to analyze tissue samples from 13 distinct regions of the human brain, including the FL, TL, PL, OL, CB, BS, THA/HT, OC, CC, VT, HIP, AN, and OB/OT. Dissected samples from each structure are pooled across the brain, digested, and analyzed via liquid chromatography‒mass spectrometry. **b** Proteomes of 13 brain regions are shown in different colors. The rings represent the number of samples (outermost ring), the total number of proteins identified in the 13 brain regions (middle), and the average counts (innermost). In the box plots, the middle bar represents the median, and the box represents the Q1–Q3 interquartile range; bars extend to 1.5 times the interquartile range to indicate the range of data, with points beyond this threshold considered outliers. FL frontal lobe, TL temporal lobe, PL parietal lobe, OL occipital lobe, CB cerebellum, BS brainstem, THA/HT thalamus/hypothalamus, OC optic chiasm, CC corpus callosum, VT ventricle, HIP hippocampus, AN amygdaloid nucleus, OB/OT olfactory bulb/olfactory tract

To investigate the proteome characteristics of regional samples from the human brain, a coarse-grained approach was initially employed to assess feasibility, with a focus on 13 gross neuroanatomical divisions: the FL, TL, PL, OL, CB, BS, THA/HT, OC, CC, VT, HIP, AN, and OB/OT (Fig. 1a). A total of 99 samples were obtained from the brains of eight individuals (Supplementary Tables 1 and 2), which were processed for MS. Relative protein abundance was analyzed in each region. These data were combined with precise anatomical localization data to create a regional proteome map of the human brain (Fig. 1b). Overall, 4,660 proteins were identified, with the majority (85–95%) present in each region: FL (4,369), TL (4,416), PL (4,391), OL (4,377), CB (4,322), BS (4,136), THA/HT (4,295), OC (4,016), CC (4,408), VT (4,320), HIP (4,448), AN (4,440), and OB/OT (4,122) (Fig. 1b, Supplementary Table 3). To ensure the reliability of the dataset, quality control assessments were performed. The median peptide count per protein was 8, and the median missing value rate was 16%, which indicated high depth and acceptable completeness of quantification (Supplementary Fig. 1a, b). The region-specific protein expression distributions (Supplementary Fig. 1c, d), intensity dynamic range (Supplementary Fig. 1e), and coefficients of variation (Supplementary Fig. 1f) further demonstrated robust measurement reproducibility across the 99 samples. The regional distribution of protein expression is shown in box plots (Supplementary Fig. 2a). PCA and PCoA score plots were used to assess sample separation (Supplementary Fig. 2b, c), and PLS-DA further illustrated grouping trends among brain region samples (Supplementary Fig. 2d).

**Fig. 2:**
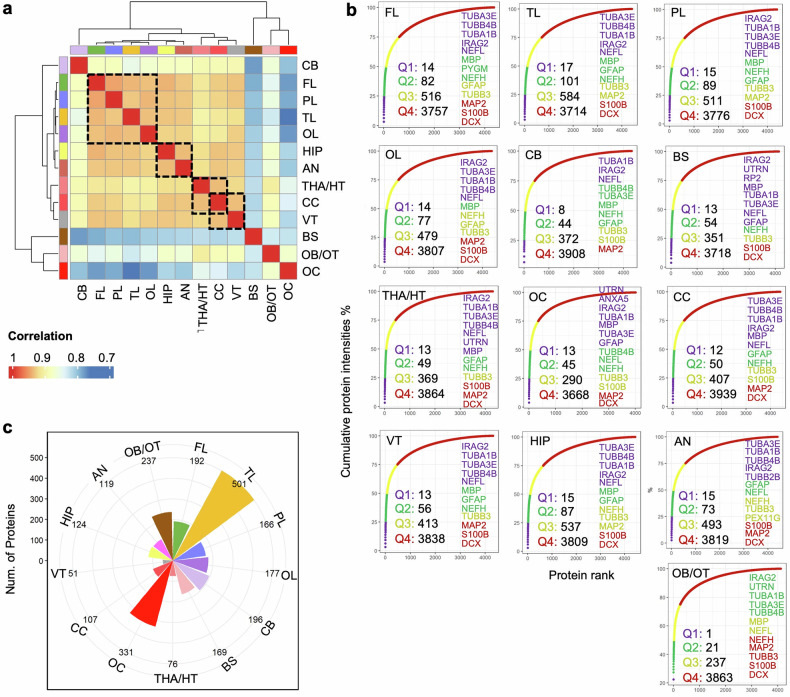
Differences in region-specific, highly expressed proteins and brain function characteristics. **a** Hierarchical clustering heatmap showing the overall relatedness of 13 brain regions. The map shows the pairwise Pearson correlation coefficients derived from region‒region correlations of the pattern of relative protein abundance. There is a tight cluster consisting of the four lobes (FL, TL, PL, and OL), a second cluster comprising HIP and AN, and a third cluster including THA/HT, CC, and VT, all of which are marked with boxes. **b** Cumulative protein abundance for 13 brain regions and the total number of proteins constituting each quartile of protein abundance (Q1: 0–25%, Q2: 25–50%, Q3: 50–75%, and Q4: 75–100%). Representative proteins of biological significance within each quartile are listed. **c** Radar plot showing the distribution of the numbers of highly expressed proteins specific to each brain region, labeled with 13 distinct colors

Pearson correlation analysis of total protein expression across various brain regions demonstrated strong correlations among several specific areas, including the four cortical lobes (FL, TL, PL, OL), between the HIP and AN, and among the THA/HT, CC, and VT. In contrast, protein expression in the remaining four regions (CB, OB/OT, BS, and OC) was significantly different (Fig. 2a). FL, TL, and PL cooperate in higher cognitive functions and information processing. Connectivity among different brain lobes is closely related to structural connectivity, particularly between the FL and PL.^17^ Furthermore, research utilizing magnetic resonance imaging (MRI) and diffusion-weighted imaging has demonstrated structural connections across lobes.^18^ For example, AD patients often exhibit neurodegenerative changes in the TL and OL, whereas disruptions in the interactions between the PL and FL can exacerbate disease symptoms.^19^ Despite the efforts of existing multiregional human brain proteomic atlases, which have compared global cortical gray and white matter, no studies have specifically investigated proteomic signatures across the four major cortical lobes or conducted lobe-specific correlation analyses.^16^ Similarly, while an optimized quantitative proteomics approach has established a cell type-resolved mouse brain secretome, that study focused primarily on cell type comparisons rather than lobar regionalization.^9^ Furthermore, although the Brodmann area-based map of the Chinese human brain cortical proteome revealed functional clustering among 29 regions and identified area-specific marker proteins, its resolution was anchored in Brodmann areas rather than lobar structures.^20^ Thus, our study extends previous multiregional proteomic research by systematically analyzing all four cerebral lobes, yielding novel insights into lobe-specific protein expression patterns.

The AN is crucial for emotional and social behavior, and the THA integrates and transmits sensory information. These two brain regions work closely to regulate stress responses and emotional memory. For example, the AN signals the HIP during the formation of emotional memories, aiding in the storage of emotionally charged experiences.^21^ Although previous studies have established neuronal connections between these two regions, no shared proteins have been confirmed.^22^ To the best of our knowledge, no prior studies have reported shared protein signatures between THA/HTs and ANs. Our findings suggest that brain regions with higher functional connectivity may also display stronger proteomic correlations. The THA/HT, CC, and VT share protein signatures. Although research supporting these shared proteins is currently lacking, these regions are anatomically adjacent within the core of the brain and participate in physiological processes, such as interhemispheric communication, fluid regulation, and homeostasis. A previous study also revealed that anatomically adjacent brain regions often share similar protein expression patterns.^16^ Conversely, CB, OB/OT, BS, and OC are functionally distinct and are involved mainly in motor coordination, olfactory processing, and basic life functions, explaining their significant differences in protein expression.

To investigate the specificity of protein abundance across different regions, proteins in each area were ranked by their relative abundance and categorized into four subsets corresponding to the quartiles (Q1: 25%, Q2: 50%, Q3: 75%, and Q4: 100%) (Fig. 2b). The findings revealed that the majority of the most highly expressed proteins (Q1 and Q2) across all brain regions are tubulins (e.g., TUBA3E, TUBB4B, and TUBB1), which are essential for the structural integrity of axons and dendrites and are crucial for neurite growth and maintenance (Fig. 1d).^23^ Other proteins consistently expressed at high levels (Q1 and Q2) include IRAG2, NEFL, NEFH, MBP, GFAP, and UTRN (Fig. 1d), all of which are vital components of the neuronal cytoskeleton. In contrast, neuronal markers such as TUBB3 (also known as TUJ1), MAP2, DCX, and S100B exhibit relatively low expression (Q3 and Q4) across all brain regions (Fig. 1d). The high expression levels of these proteins minimize the likelihood of technical artifacts. Despite those common proteins across all regions, there are still differences in protein abundance (e.g., PYGM in the FL, RP2 in the BS, UTRN in the OL, THA/HT, BS, and PEX11G in the AN). Research has shown that glutamatergic activity and energy metabolism are reduced in the prefrontal cortex of patients with schizophrenia, whereas treatment with the NMDAR antagonist ketamine in a psychosis model mice increases PYGM levels.^24^ Although the presence of the UTRN in the brain has not been as widely discussed as in muscle tissues, its role in maintaining cellular structure could be essential for regions such as the THA and BS, which are integral for sensory processing and motor coordination.^25^ Studies on regional protein expression, such as those from the HPA, provide insights into how proteins such as UTRNs are distributed across different brain regions. However, further research may be needed to understand the mechanisms behind its expression pattern in the brain. PEX11G is highly enriched in AN, which aligns with our observations.^26^ However, the current literature lacks detailed studies focused explicitly on PEX11G function in the brain, suggesting that this may be an area worth further exploration. Overall, this region-resolved proteomic profiling system is robust for identifying unique proteomic components in the 13 brain regions. We suggest that these variations in abundance may provide potential alternative target molecules for the design of immunohistochemical experiments to distinguish between different brain regions. However, highly abundant proteins may not reflect the specific function of a given brain region. Further research is needed to identify region-specific, highly expressed proteins to explore the unique functions of these areas.

On the basis of proteomic characteristics, we propose a three-module conceptual framework: the cortical integration module (FL, TL, PL, OL), the limbic-relay network (AN, HIP, THA/HT), and the midline regulatory axis (THA/HT, CC, VT, OC). These modules are defined by shared protein expression patterns and anatomical contiguity. In the following sections, we focus on each module to explore its distinct proteomic signatures and functional implications.

### Functional differences and the brain region-protein-function network

To investigate brain region-specific functions, a total of 2,446 region-specific, highly expressed proteins were analyzed from 13 brain areas: FL (192), TL (501), PL (166), OL (177), CB (196), BS (169), THA/HT (76), OC (331), CC (107), VT (51), HIP (124), AN (119), and OB/OT (237) (Fig. 2c, Supplementary Fig. 3a, Supplementary Table 4). Notably, the TL region presented the greatest number of region-specific, highly expressed proteins, followed by the OC region. In contrast, the VT has the lowest number. The high abundance of specific proteins in the TL region may be linked to its critical functions in visual processing and information integration. As a central area for visual information processing, the protein characteristics of the OC also reflect the specific demands of visual function. The high protein specificity in the TL region further suggests molecular specialization associated with complex visual and cognitive processing.

Hierarchical clustering and GO term analyses were conducted to predict the distinct functions of these regions (Supplementary Fig. 3b). Each brain region is enriched with region-specific, highly expressed proteins that collectively underlie common functional processes, including cellular metabolism (e.g., respiration and ATP production), neurotransmission (e.g., synaptic vesicle endocytosis and neurotransmitter secretion), cellular structure and development (e.g., neuronal projection development and axonogenesis), and intracellular transport.

BS is considered to play important roles in energy metabolism and neuronal function and appears to respond effectively to oxidative stress. A previous murine study also revealed that brainstem ciliary signaling critically contributes to the regulation of glucose balance, lean mass, and autonomic function.^27^ THA/HT regulates behavior and hormone metabolism, which may contribute to endocrine functions. Experimental evidence has demonstrated that the activation of medial amygdala-ventromedial hypothalamic circuits under acute stress drives behavioral adaptations while concurrently modulating glucose homeostasis.^28^ Moreover, comparative zebrafish and mouse studies revealed that hypothalamic neurons integrate metabolic hormones, such as growth hormone, to regulate feeding behavior and maintain energy balance.^29^ The CC is important for cortical development and oligodendrocyte maturation, potentially supporting myelination and rapid signal conduction through myelinated nerve fibers. MRI studies have revealed a link between CC myelin content and cognitive function, with changes associated with disorders such as multiple sclerosis.^30^

The HIP is considered to play a critical role in learning and memory and is involved in “dendritic spine development” and “regulation of dendrite morphogenesis”, suggesting its potential contribution to neuronal connectivity and plasticity. Experimental studies using rats after discharge-induced seizures have demonstrated that hippocampal dendritic spines undergo shrinkage and structural remodeling, accompanied by synaptic weakening and alterations in AMPA receptors, highlighting the role of HIP in dendrite morphogenesis and synaptic plasticity.^31^ The function of the VT is underexplored, whereas this research suggests unexpectedly high expression of biological processes. Ependymal cells in the VT likely support neural development, as indicated by “glial cell differentiation” and “positive regulation of gliogenesis”. The choroid plexus may require efficient energy metabolism for cerebrospinal fluid (CSF) generation, as suggested by processes linked to “precursor metabolites and energy”. The cells within the VT may influence neuronal connectivity, as implied by “synaptic vesicle localization” and “synapse assembly”. Finally, the VT may play roles in embryonic development and physiological regulation, as indicated by the “regulation of cell cycle phase transition” and “regulation of systemic arterial blood pressure”. While the TL has the most and VT the fewest region-specific proteins, the understudied midline regulatory axes (THA/HT, CC, VT, OC) exhibit unexpectedly rich functional activity, suggesting specialized roles in neurodevelopment and physiological homeostasis. Single-cell transcriptomic analyses of the developing ventricular zone of mice demonstrated that ependymal cells and neural stem cells arise from radial glial cells, supporting neural development through glial cell differentiation and gliogenesis.^32^ These studies further highlight the potential roles of VT cells in neuronal connectivity, as indicated by synapse assembly and synaptic vesicle localization, and in physiological regulation, including cell cycle control and systemic blood pressure. Overall, experimental evidence from developmental and lineage-tracing studies suggests that the understudied VT, as part of the midline regulatory axis, plays specialized roles in neurodevelopment and homeostatic regulation.

**Fig. 3:**
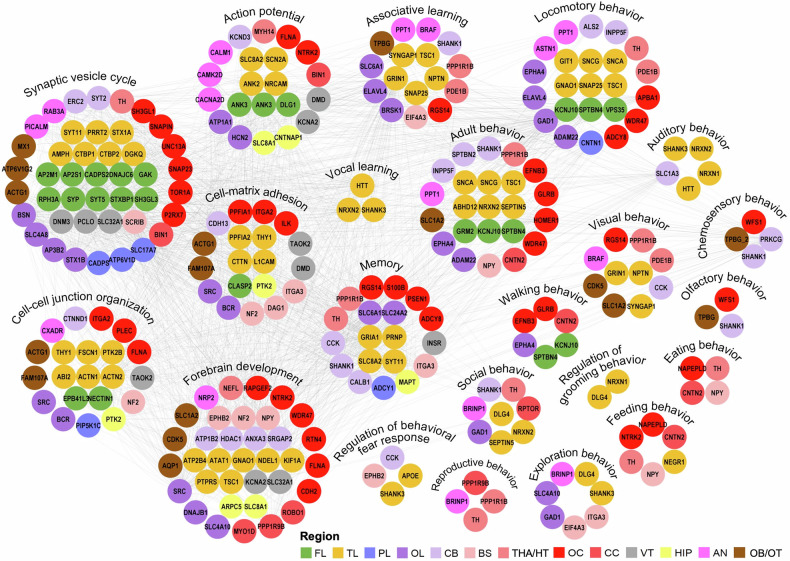
Protein‒protein interaction network of region-specific, highly expressed proteins constructed via the STRING database. This network retains specific highly expressed proteins with documented interactions organized by functional units. Several molecules are involved in multiple brain functions and behavioral characteristics

A brain region‒protein‒function network visually represented the connections between each biological process and functional cooperation among these areas (Fig. 3). These functions can be broadly classified into three major categories: fundamental processes, sensorimotor functions, and advanced, evolutionarily recent functions. Fundamental processes include synaptic vesicle cycling, cell‒cell junction organization, cell‒matrix adhesion, and action potential generation. Sensorimotor functions encompass motor control and sensory/perceptual processing, such as walking behavior; locomotory behavior; and visual, auditory, chemosensory, and olfactory responses. Advanced processes are grouped into several categories: brain development and learning/memory (forebrain development, associative learning, vocal learning, and memory), feeding and nutritional regulation (eating and feeding behaviors), and emotional and social behavior regulation (adult behavior, fear response regulation, grooming behavior regulation, reproductive behavior, social behavior, and exploratory behavior).

Fundamental processes involve nearly all brain regions. Among them, the TL contributes the greatest diversity of region-specific, highly expressed proteins, followed by the OC, both of which markedly surpass other cortical areas. Although the THA/HT and CC contribute relatively few proteins, they include key regulators such as TH (THA/HT) and BIN1 (CC), which are involved in synaptic vesicle cycling. BIN1 (CC) and MYH14 (THA/HT) also participate in action potential generation. The VT—despite being previously undercharacterized—contributes a distinct set of region-specific proteins, including DNM3, SLC32A1, and PCLO, which are implicated in synaptic vesicle cycling; DMD and TAOK2 are associated with cell‒cell junction organization and cell‒matrix adhesion, whereas DMD and KCNA2 are involved in action potential generation. Our findings suggest that the midline regulatory axis is more active than traditionally appreciated.

With respect to sensory function, previous studies emphasized the connections between the OC and TL in visual behavior, with supporting roles of the AN, THA/HT, and CB; the OB/OT governs olfactory and chemosensory behaviors.^33–35^ Our findings indicate that the OB/OT may also participate in visual processing (CDK5, SLC1A2), and the OC is suggested to be involved in olfactory and chemosensory behaviors (WFS1).^36–38^ Although previous studies have reported the roles of these proteins, their enrichment in these particular brain regions has not, to our knowledge, been documented. Some studies have shown that the integrated processing of olfactory and visual information enhances the ability to detect potential threats and locate food in animals.^39^ We provide the first proteomic evidence for functional overlap between the OB/OT and OC, offering theoretical support for the concept of cross-modal integration. We reveal the extensive involvement of the CB in sensory and perceptual processing (SHANK1, PRKCG, CCK),^40–44^ providing the first proteomic evidence for the dual role of the CB in both motor and sensory functions. Overall, the midline regulatory axis, particularly the VT, may contribute key proteins to fundamental processes despite its limited involvement in advanced functions. Our findings also suggest that the CB is a dual-function region involved in both motor and sensory regulation at the proteomic level.

For advanced functions, nearly all brain regions are involved in adult behavior. Traditionally, forebrain development and associative learning are linked to the TL and AN, whereas memory is related to the TL and HIP, and vocal learning primarily involves the TL. Our study revealed that in addition to the TL, AN, and HIP, the OL, THA/HT, BS, OB/OT, and CC may also express region-specific, highly expressed proteins relevant to these functions (CCK, CDH2, NF2, PPP1R9B), with an unexpectedly significant contribution from the OC.^45,46^ Although studies on CDH2 and NF2 have confirmed the roles of these proteins in learning, they have focused primarily on the HIP and FL. In contrast, we detected CDH2 expression in the OC and NF2 expression in the BS. Additionally, PPP1R9B is highly specific for expression within the CC and THA/HT. Furthermore, the THA/HT, CC, and CB are implicated in social behavior (PPP1R1B, RPTOR, SPTBN2), despite limited evidence or ongoing debate regarding their roles.^47–49^ Reproductive behavior is associated not only with the AN but also with the THA/HT and CC (TH, PPP1R9B). Exploratory behavior is traditionally linked to TL, OL, and AN, with relatively little research on BS;^50^ our findings offer new evidence for the role of BS in exploratory behavior (ITGA3, EIF4A3). The regulation of the behavioral fear response is related to the TL, CB, and BS (EPHB2, APOE, SHANK3, CCK), which we substantiate.^51^ However, previous studies on fear responses have typically focused on the AN. In this study, we identified proteins found in the AN that are also specifically expressed in the TL, CB, and BS, suggesting novel research directions for further investigation.^52,53^ DLG4 has been associated with compulsive behaviors in the mouse cortex; however, we have specifically narrowed the focus to the TL.^54^ In terms of feeding and nutritional regulation, current research confirms that multiple brain regions, primarily involving the THA/HT, cooperate in regulating eating behavior (TH),^55,56^ and the TL, THA/HT, and BS predominantly govern feeding behavior (NTRK2, NPY).^57–59^ Reports suggest that hunger could affect the CC.^60^ All these findings are validated within this network. Overall, previous studies have shown that lower brain structures, such as the BS and CB, are rarely associated with advanced social behaviors; however, our findings challenge this perspective. On the basis of the proposed three-module framework, the midline regulatory axis is actively expressed in gliogenesis, synaptic assembly, energy metabolism, and embryonic development, indicating a potential role in supporting neurodevelopment and higher-order functional regulation, particularly in maintaining brain structural stability and social behavior. The BS and CB regions, traditionally viewed as lower-order structures, exhibit unexpected functional complexity, suggesting critical involvement in integrating sensory inputs and coordinating behavioral outputs.

### Cell composition-specific characteristics of brain regions

**Fig. 4:**
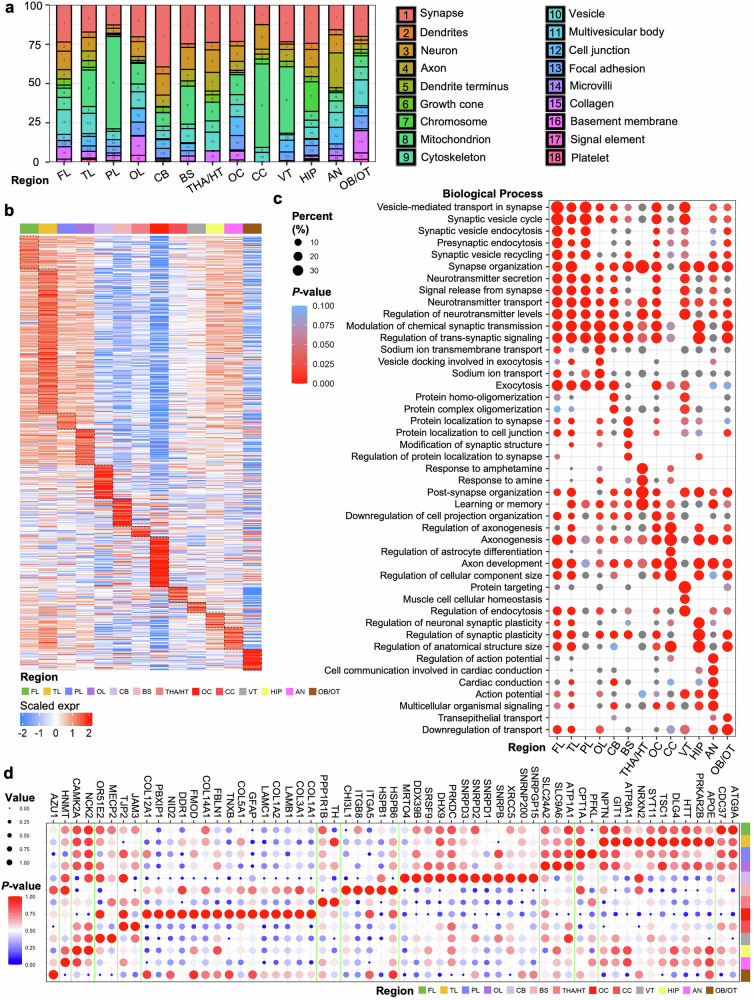
Cellular component analysis of brain regions and functional profiling of region-specific, highly expressed proteins in neurons. **a** Cellular component analysis of proteins from the 13 brain regions. The relative abundance of region-specific, highly expressed cellular components of each region is shown in a percentage stacked column chart. Gene Ontology (GO) CC annotations were retrieved from the OrgDb database via ClusterProfiler. Each color with a number represents one cellular component. **b** Heatmap of the proteins differentially expressed in neurons across 13 brain regions. Coexpression analysis of the protein modules obtained from 13 brain regions revealed their proteome specificity. The heatmap displays z scored normalized intensities of significantly differentially expressed proteins on the basis of unsupervised hierarchical clustering. The colors correspond to protein identity, with red and blue boxes indicating proteins with increased and decreased abundance, respectively. **c** Cell signaling pathways involving region-specific, highly expressed proteins in neurons across different brain regions. Circles of varying sizes represent the percentage of proteins included in each biological process. Boxes in the red gradient indicate the degree of enrichment on the basis of *p*-values. **d** Region-specific, highly expressed proteins in neurons contributing to important brain function regulation

We further analyzed the cellular components associated with the region-specific, highly expressed proteins in these brain regions (Fig. 4a). Our focus was also on neuronal cells, where we examined their highly expressed biological processes (Fig. 4b, c); for these biological processes, we identified the specific proteins involved (Fig. 4d). Additionally, we separately analyzed the highly expressed biological processes within dendrites, axons, and vesicles (Supplementary Fig. 4–6). The distribution patterns of cellular components across different brain regions are consistent with the specific functional demands of each area (Fig. 4a). The high abundance of mitochondria in the PL, CC, and VT suggests that these regions may be metabolically active. Extensive connectivity between the PL and FL may underlie higher-order cognitive processes, and age-related metabolic decline in these areas may contribute to cognitive disorders such as AD. As supported by our data, the CC and VT appear to be associated with synaptic vesicle cycling and action potential generation, whereas the VT also participates in cell‒cell junction organization and cell‒matrix adhesion. Mitochondrial enrichment in these regions implies that processes such as interhemispheric communication, CSF circulation, and maintenance of blood‒brain barrier (BBB) integrity are likely energetically demanding. The predominance of synapses in the FL, OL, CB, THA/HT, OC, HIP, and AN suggests that these regions are involved in complex neural signal transmission and integration. The high presence of axons in OB/OT indicates the role of these regions in long-distance signal transmission, whereas the abundance of vesicles in AN may be linked to neurotransmitter release and regulation. As noted above, our findings indicate a potential shared sensory processing network between the OB/OT and OC, both of which possibly contribute to visual and olfactory functions. The elevated expression of synaptic and axonal proteins suggests that extensive connectivity is essential for cross-modal integration, which may be vulnerable to synaptic loss. Although traditionally considered a motor center and a visual hub, respectively, the CB and OC appear to be involved in higher-order functions. Their synaptic protein levels, just below those of the FL and OL, support broader cognitive and integrative roles than previously assumed. The elevated levels of cell junctions, focal adhesions, microvilli, collagen, and the basement membrane in OLs, ANs, and OCs reflect the importance of these components in maintaining structural integrity and functional connectivity through physical and signaling interactions. These components are proposed to provide mechanical support, facilitate information transfer, and regulate cellular functions.^61^

We first examined the protein expression profiles of neurons across different brain regions (Fig. 4b), followed by an analysis of the upregulated biological processes in neurons (Fig. 4c). Neurons in the cortical integration module show similar upregulation of synaptic vesicle cycling, synapse organization, signaling, and neurotransmitter regulation. THA/HT may play central roles in information transmission, emotional regulation, and behavior control and is modulated by neurotransmitters such as dopamine, norepinephrine, and serotonin—targets of amphetamine and amine compounds. In the OC and CC, upregulated processes included negative regulation of cell projection organization, axonogenesis, astrocyte differentiation, axon development, and protein targeting. The CC likely depends on robust axonogenesis and astrocytic support for precise and efficient signal transmission. Although not a classic connectivity hub, the OC appears to display similar molecular features, suggesting broader integrative roles consistent with its extensive functional involvement (Fig. 3). The cells in the VT, including ependymal cells, rely on specific proteins for synthesizing and secreting CSF. The high level of protein targeting indicates the need for precise protein synthesis and secretion. Endocytosis, which involves membrane remodeling and internalization, may be vital for the cellular uptake of external substances. In the HIP, we observed high expression of proteins involved in regulating synaptic plasticity. Synaptic plasticity, particularly long-term potentiation (LTP) and long-term depression, is thought to be essential for hippocampal circuits.^62^

Focusing on neuron-specific proteins across the cortical integration module, we identified a highly consistent protein profile (Fig. 4d). Key proteins include CDC37 (kinase chaperone),^63^ ATG9A (autophagy and metabolic balance),^64^ APOE and DLG4 (Alzheimer’s disease), HTT (Huntington’s disease),^65–67^ and TSC1 (mTOR inhibition, tuberous sclerosis).^68,69^ SYT11, NRXN2, NPTN (neurotransmitter release and plasticity),^70–72^ MECP2 (gene regulation, Rett syndrome),^73^ and NCK2 (cytoskeletal remodeling)^74^ were also enriched. High CAMK2A expression reflects its role in LTP, learning, and memory.^75^ Collectively, the cortical integration module and its associated proteins may represent a molecularly vulnerable network in neurodegenerative disorders, with synaptic dysfunction and metabolic imbalance serving as common pathological features. With respect to the limbic-relay network, in THA/HT, TH and PPP1R1B regulate dopamine synthesis and signaling, are critical for emotional and autonomic control, and have been implicated in schizophrenia.^76,77^ For the midline regulatory axis, in the OC, collagens and laminins maintain structural stability, whereas GFAP, fibronectin, and other ECM proteins support astrocytic and tissue repair functions.^78^ JAM3 and TJP2 in the CC contribute to cell adhesion and blood‒brain barrier maintenance.^79^ Collectively, these features reinforce the concept that the midline regulatory axis may play a fundamental role through ECM proteins in structural coordination, barrier integrity, and stress responses, potentially forming the anatomical and molecular backbone for maintaining cerebral stability and homeostasis.

Biological processes in axons, dendrites, and vesicles were examined. In axons, structural maintenance and signal transduction are highly regulated (Supplementary Fig. 4b). In THA/HT, enriched processes include regulating presynaptic cytosolic calcium, responding to corticosterone and mineralocorticoids, and establishing intermediate filaments, which may indicate endocrine sensitivity. Dendrites across regions were enriched in neurotransmission, morphogenesis, plasticity, and membrane transport (Supplementary Fig. 5b). THA/HT dendrites also respond to amphetamine, alkaloids, and amines, suggesting a link between neurotransmitter response and reproductive behavior. Vesicles show widespread upregulation of synaptic transmission, neuronal signaling, and metabolism (Supplementary Fig. 6b). In THA/HT vesicles, processes such as late endosome‒vacuole transport, EGF receptor signaling inhibition, and multivesicular body assembly are prominent. OC vesicles are enriched in protein localization maintenance, ceramide transport, and phagocytosis. In VT, receptor-mediated endocytosis and membrane protein targeting are increased. Efficient vesicle transport and synaptic cycling are essential for neuronal function and metabolic waste handling.^80^ The axons, dendrites, and vesicles within the midline regulatory axis presented distinct patterns of enriched pathways, in clear contrast to those in the cortical integration module (Supplementary Fig. 4b, 5b, 6b). This may suggest notable heterogeneity among its constituent structures, along with coordinated and nuanced information exchange across regions.

### Capture of protein signatures across brain regions

**Fig. 5:**
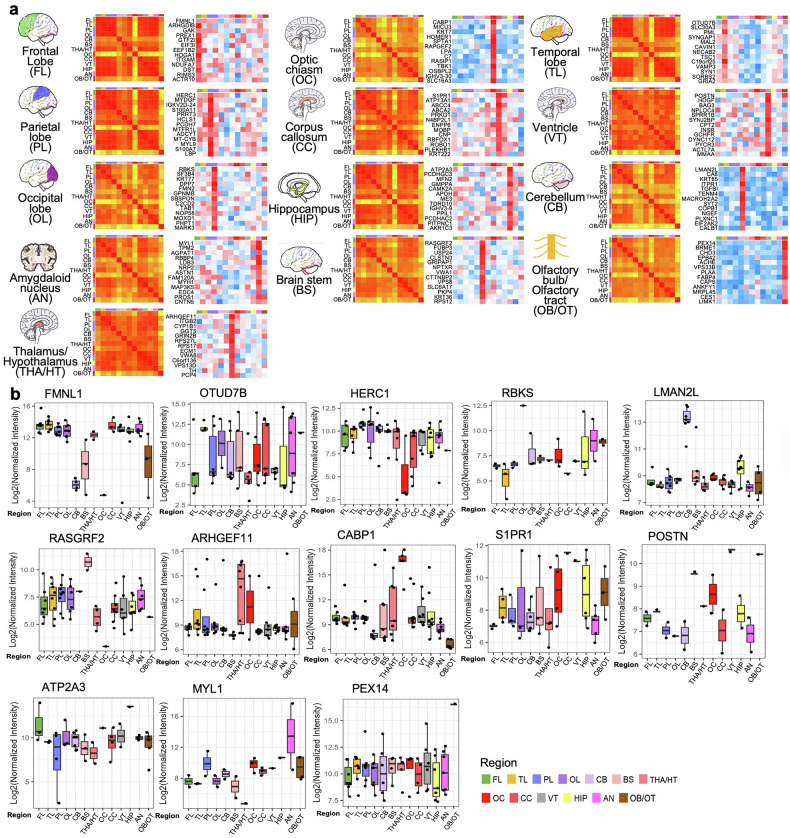
Representative region-specific, highly expressed protein profiles for each brain region and distinctions among profiles across brain regions. **a** Schematic diagram, correlation analysis, and protein expression heatmap. The heatmap depicts Pearson correlation coefficients between pairs of 13 brain regions on the basis of log2 transformed and normalized abundance of the region-specific, highly expressed proteome of each region. Additionally, the top 13 highly expressed region-specific proteins for each brain region and their expression levels in other regions are presented. The fold changes in proteins for each brain region were calculated in comparison with the average expression of samples from the remaining 12 brain regions. **b** Representatives of region-specific, highly expressed proteins for each brain region. The figure presents the specific protein with the highest expression for each brain region and its expression levels in other regions

The 13 proteins with the most significant changes in expression across each brain region were analyzed (Fig. 5a). We also identified the top region-specific proteins in each region, highlighting their expression profiles (Fig. 5b). The midline regulatory axis exhibits region-specific protein expression profiles encompassing neuronal signaling, cytoskeletal organization, energy homeostasis, and neuroprotection, suggesting a broader regulatory and protective role beyond its traditional function as a structural conduit. In particular, S1PR1, which is highly expressed in the CC, plays an expanded role in signal transduction rather than serving solely as a relay center. VT is associated with elevated expression of POSTN, a neuroprotective and developmental protein, highlighting its potential involvement in neural maintenance. Similarly, OB/OT has high levels of PEX14, indicating that it has underappreciated central regulatory functions. The OC strongly expresses CABP1, suggesting a more active role in sensory-integration processing rather than being a passive terminal structure. This proteomic landscape reveals previously unrecognized functional diversity within the midline regulatory axis.

An analysis was performed to identify proteins that are commonly and specifically highly expressed across the four cortical lobes (i.e., the cortical integration module) relative to noncortical brain regions (Supplementary Fig. 7a). This distinction is important because proteins simply shared across all four lobes are numerous and lack regional specificity. Therefore, the focus was placed on the subset of proteins that are both shared among and enriched in the four lobes compared with noncortical regions, representing cortical lobe-specific molecular signatures. As shown in Supplementary Fig. 7a, the Venn diagram reveals 26 proteins (3.5%) that are commonly and highly expressed in all four lobes. These include SYN3, PALM, CLSTN1, GNAO1, ACTN1, HK1, ATP2B1, RGS7, SIRPA, CLTC, ADCY1, PAK1, EIPR1, STXBP5, SLC27A4, CADM2, DPP10, BRSK1, PGAM5, LYSMD1, ATG5, WDR13, LYRM4, PLCB1, CAMSAP3, and EPN1. Together, these proteins form a shared cortical molecular framework distinct from that of noncortical areas.

To assess their functional relevance, pathway enrichment analysis was conducted (Supplementary Fig. 7b). The commonly enriched biological processes included the regulation of transsynaptic signaling, vesicle-mediated transport, the regulation of metal ion transport, the regulation of microtubule cytoskeleton organization, cell–cell adhesion, behavior, learning or memory, vascular processes in the circulatory system, neuron projection development, and cognition. Among these, the regulation of transsynaptic signaling and vesicle-mediated transport showed the strongest enrichment, along with additional processes central to neuronal communication and increased cognitive functions.

These functional categories align closely with hallmark cortical activities such as synaptic integration, circuit-level plasticity, and complex information processing, indicating that the shared protein signature represents a conserved functional module across cortical lobes. Importantly, dysregulation of several proteins within these pathways has been implicated in cortical dysfunction–related neurological and psychiatric disorders. For example, ADCY1,^81^ PLCB1,^82^ and RGS7^83^ participate in GPCR-mediated synaptic signaling and are associated with schizophrenia, metabolic disorders, psychostimulant addiction, and cognitive impairment. CLSTN1 and CADM2, which are involved in synapse organization and connectivity, have been linked to autism spectrum disorders and neurodevelopmental delay.^84^ HK1^85^ and ATP2B1,^86^ which regulate neuronal energy homeostasis and calcium dynamics, are connected to epileptic susceptibility and synaptic hyperexcitability. PAK1^87^ and BRSK1^88^ both modulate cytoskeletal dynamics and synaptic plasticity. Their involvement in inflammation-mediated changes in neuronal excitability and cell survival has been linked to the pathogenesis of seizure activity.

Thus, these commonly enriched cortical-lobe proteins collectively highlight a shared synaptic signaling and vesicle transport regulatory module that likely underlies cortical specialization and coordinates increased cognitive function while also providing mechanistic links to disease vulnerability when dysregulated.

FMNL1 is a cytoskeletal regulatory protein involved in actin filament organization and cell motility. Notably, FMNL1 functions in addition to normal brain physiology and plays a critical role in glioblastoma multiforme.^89^ In our dataset, FMNL1 was markedly more highly expressed in the four lobes, as well as in the VT, CC, HIP, and AN, whereas its expression was particularly low in the CB, OC, and OB/OT (Fig. 5b). To experimentally validate these proteomic findings, we performed additional immunohistochemical staining, which confirmed that the region-specific expression pattern of FMNL1 was consistent with the proteomic data (Supplementary Fig. 8a).

Given the core role of FMNL1 in actin cytoskeleton remodeling, its high expression in the cerebral cortex likely supports the structural and functional plasticity of cortical neurons. The cortex is the center for increased brain functions, which depend heavily on dynamic actin rearrangements to maintain synaptic formation, dendritic spine remodeling, and neuronal connectivity. Similarly, high FMNL1 expression in the HIP and AN suggests that it may modulate synaptic plasticity linked to memory encoding and emotional regulation. The VT and CC, which are responsible for CSF homeostasis and interhemispheric signal transmission, respectively, may rely on FMNL1-driven cytoskeletal dynamics to sustain ependymal cell integrity and commissural fiber growth.

Subsequent integration of these results with the previously established brain region‒protein‒function network (Fig. 3) suggested that proteins with the most significant expression changes in each region may not fully represent those with substantial biological relevance. Several proteins, although not ranked among the top differentially expressed proteins, demonstrate consistent involvement across multiple biological processes within specific regions. For example, in VT, proteins such as DMD, KCNA2, DNM3, SLC32A1, and TACR2 appear to be involved in synaptic function, ion transport, and neurotransmission regulation. In the CC, CDKN2A and PPP1R9B contribute to signal integration and synaptic organization. The OC features high expression of GLRB, EFNB3, and WFS1, all of which are linked to sensory processing and synaptic signaling. Similarly, CDK5 and TPBG in the OB/OT are associated with neurodevelopment and synaptic remodeling. Notably, a large proportion of key functional proteins within the midline regulatory axis are localized to synapses, underscoring the potential role of synaptic integrity in mediating its regulatory functions. This synaptic dependence also suggests that synaptic vulnerability may underlie the pathophysiology of many midline regulatory axis-related neurological disorders.

The Limbic-Relay Network has been proposed to serve as a key hub for emotion and cognition integration and transmission. In our analysis of THA/HT, PPP1R1B and TH were identified as key functional proteins. TH encodes tyrosine hydroxylase, the rate-limiting enzyme in catecholamine biosynthesis, and plays a crucial role in dopamine-mediated motor control, reward processing, and mood regulation. Its deficiency is associated with neurodegenerative disorders such as Parkinson’s disease.^90^ Within the AN, BRINP1, PPT1, and BRAF are involved in lipid metabolism and intracellular signaling, with potential implications for emotional and social behavior regulation.^91^ The cortical integration module is enriched predominantly in proteins involved in learning, memory, and synaptic plasticity (SYNGAP1, SYN1, ADCY1, FMN2, NOP58), which is consistent with its role in higher-order cognitive processes.^92–97^

### Enhanced resolution of synaptic and metabolic functions via proteomic profiling

**Fig. 6:**
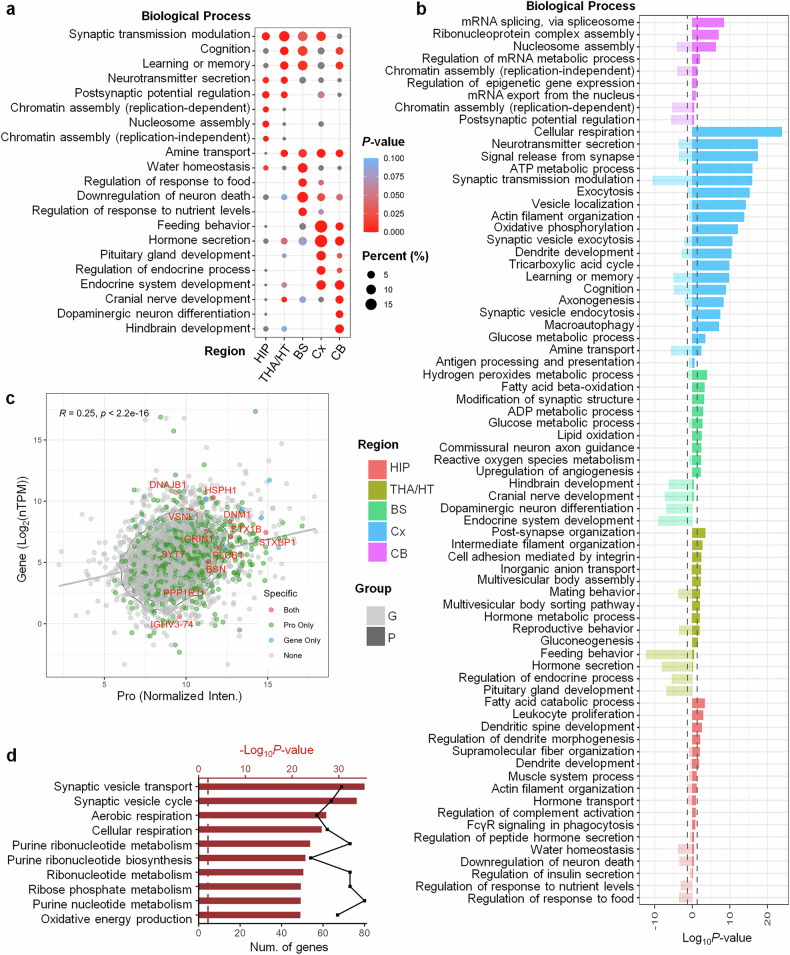
Comparison of the results of pathway analysis of the human brain via proteomics and transcriptomics. **a** Pathways associated with region-specific, highly expressed proteins in the human brain according to the Human Protein Atlas (HPA) database. Circles of different sizes represent the percentage of proteins included in each biological process. Boxes in the red gradient indicate the degree of enrichment on the basis of the *p*-value. **b** Comparison of the cellular signaling pathways associated with the region-specific, highly expressed proteins identified in five brain regions via both transcriptomic and proteomic analyses. Different colors represent different brain regions, with high saturation indicating conclusions derived from proteomics and low saturation representing those from transcriptomics on the basis of the HPA database. **c** Scatter plot of region-specific, highly expressed proteins identified in the lobes (FL, TL, PL, and OL) via transcriptomic and proteomic analyses. Red indicates proteins identified by both analyses, green indicates proteins identified solely by proteomics, and blue indicates proteins identified only by transcriptomics. Gray represents genes and proteins without established transcriptional connections. **d** Pathways associated with proteins identified only by proteomic analysis in the lobes (FL, TL, PL, and OL). Line graphs depict the number of proteins involved in each cellular signaling pathway, while bar graphs represent the enrichment significance of biological processes, expressed as the -log *p* value

To assess the reliability of proteomic profiling in the human brain, we integrated transcriptomic data from the HPA database for cross-validation and comparative analysis (Fig. 6). Five brain regions were included: the HIP, THA/HT, BS, cortex (Cx, corresponding to FL, TL, PL, OL, OB/OT in our data), and CB (Fig. 6a). In the BS, transcriptomics identifies neurodevelopmental processes such as hindbrain development, cranial nerve formation, dopaminergic neuron differentiation, and endocrine development. In contrast, proteomics highlights additional pathways, including hydrogen peroxide metabolism, fatty acid beta-oxidation, synaptic remodeling, ADP and glucose metabolism, lipid oxidation, commissural axon guidance, ROS metabolism, and angiogenesis regulation. These results suggest that proteomic data may provide a more detailed view of cellular metabolism and structural plasticity, including key processes such as energy homeostasis and synaptic adaptation (Fig. 6b). In THA/HT, proteomics uniquely reveals postsynaptic organization, intermediate filament regulation, integrin-mediated adhesion, anion transport, multivesicular body assembly/sorting, hormone metabolism, and gluconeogenesis. Moreover, transcriptomics can be used to identify endocrine-related functions such as feeding behavior, hormone secretion, and pituitary development. These differences may partly reflect the impact of posttranslational modifications, which affect protein detectability and function but are not captured at the RNA level. The proteomic emphasis on functional protein activity may explain our discovery of previously unrecognized metabolic, developmental, and homeostatic features within the midline regulatory axis.

We further mapped gene‒protein correspondences to examine overlap and divergence between the datasets (Fig. 6c, Supplementary Fig. 9a). Proteomics consistently identifies more region-specific proteins, while a subset was detected by both approaches, and a few appeared only via transcriptomics. The functional enrichment of proteins identified exclusively by proteomics clustered in the vesicle-mediated transport, synaptic vesicle cycling, aerobic and cellular respiration, and purine metabolism pathways (Fig. 6d). These pathways are suggestive of cellular energy transformation and synaptic communication, processes that may be fundamental to brain activity and metabolic regulation. In summary, proteomics and transcriptomics appear to offer complementary views of brain physiology: the former reflects protein-level function and activity, whereas the latter captures gene regulation. Integrating both layers may enhance our understanding of region-specific cellular mechanisms in the human brain.

## Discussion

This study represents a comprehensive proteomic mapping of functional regions across the human brain, providing a detailed analysis of region-specific protein characteristics and their functional implications. We analyzed 13 distinct brain regions from postmortem human samples and identified many proteins commonly shared across regions. To explore human-specific advanced intelligence, our research focused on human samples, enhancing the level of evidence compared with in vitro neuron experiments, mouse models, and MRI. This proteomic analysis offers a novel approach to understanding the diversity and specificity of brain functions across multiple regions, providing a higher resolution of normal brain physiology for future studies.

Previous work by Guo *et al*. generated a global multiregional proteomic map of the human cerebral cortex on the basis of Brodmann areas;^20^ however, our study differs in that we systematically analyzed the cerebral lobes, subcortical nuclei, major white matter tracts, cerebellum, brainstem, and ventricles, thereby offering complementary insights into protein expression patterns. Moreover, our findings are consistent with and extend those of previous studies. For example, a mouse brain secretome model revealed high expression of cell adhesion, metabolic process, neuron projection, cellular complex, and synaptic signaling-related proteins in neurons.^9^ Our results confirmed these observations and further localized these functions to specific brain regions (Fig. 4c). We found that synaptic signaling was highly expressed in neurons across nearly all regions, particularly within the four lobes, whereas cell adhesion was predominantly enriched in the VT, possibly related to the maintenance of BBB integrity. Together, our data highlight interregional molecular differences that build upon and refine previous findings.

Another study by Tüshaus et al.^16^ characterized protein functions across major brain structures, including white matter nuclei, gray matter, and CB. Consistently, we also observed region-specific enrichment of IGHV family proteins in the HIP (Fig. 5a), supporting their results. This study also analyzed four principal brain cell types—neurons, astrocytes, microglia, and oligodendrocytes—and our findings similarly confirmed the high proportion of synaptic components in cortical neurons (Fig. 4a). Furthermore, we focused on components rarely emphasized in previous works, including cell junctions, focal adhesion, the basement membrane, and collagen, which are fundamental to maintaining brain homeostasis. In addition, our study further analyzed functional enrichment specifically within neuronal compartments—dendrites, axons, and vesicular structures—representing an additional layer of resolution not achieved in prior studies (Fig. 4c, Supplementary Fig. 4–6).

Guo *et al*. constructed a human cerebral cortex proteomic atlas and reported that Brodmann areas with similar protein expression profiles often share similar functions, even when they are not spatially adjacent.^20^ For example, the temporal pole showed protein patterns resembling both the sensorimotor cortex and Wernicke’s area. Overall, their study demonstrated that the proteome better represents cortical functional differentiation. In agreement with these observations, our study also revealed molecular associations between spatially distant brain regions. The OB/OT may contribute to visual processing, whereas the OC appears to be involved in olfactory and chemosensory behaviors, suggesting potential cross-modal interactions. Whereas Guo et al. grouped cortical regions into six clusters on the basis of proteome-defined functions, we propose a three-module framework, which highlights the previously underrecognized midline regulatory axis, offering new insight into the molecular and functional organization of the human brain.

Notably, the TL region has the greatest number of region-specific, highly expressed proteins, underscoring its significant role in brain functions such as auditory and visual processing, memory, and sensory integration. The CC may play a critical role in cortical development and oligodendrocyte maturation, contributing to rapid signal conduction through myelinated nerve fibers. VT, an underexplored region, shows unexpectedly high expression in biological processes, suggesting its importance in neural development, as indicated by elevated levels of glial cell differentiation and positive regulation of gliogenesis. Interestingly, the OB/OT may participate in visual processing, whereas the OC appears to be involved in olfactory and chemosensory behaviors, suggesting possible interactions between these regions. The OC, although not a typical neuronal connection hub, shares characteristics with the CC, hinting at the potential for more extensive connectivity. Moreover, we suggest the extensive involvement of CB in sensory and perceptual processing, emphasizing the functional complexity across these brain regions.

In this study, guided by hierarchical clustering of regional protein expression correlations (Fig. 2a), we propose a novel three-module framework for human brain regional organization: the cortical integration module (FL, TL, PL, OL), the limbic-relay network (AN, HIP, THA/HT), and the midline regulatory axis (THA/HT, CC, VT, OC). In line with this framework, converging evidence supports functional interconnections among the THA/HT, CC, VT, and OC. Recent anatomical studies in primates have identified thalamic commissures as novel cross-hemispheric pathways, underscoring the THA as a hub complementing CC-mediated interhemispheric communication.^98^ The suprachiasmatic nucleus provides further evidence of HT integration of photic inputs from the OC to regulate circadian rhythms.^99^ Developmental studies also emphasize these linkages: cases of hypothalamic hamartoma with concomitant callosal agenesis suggest developmental interplay between THA and the CC,^100^ and fetal diffusion MRI has shown synchronous growth and convergence of the thalamocortical and callosal pathways during early gestation, establishing a substrate for later functional integration.^101^ From a pathological perspective, third ventricle enlargement in multiple sclerosis has been closely linked to THA damage and cognitive decline, highlighting the functional relevance of thalamo-ventricular interactions.^102^ In patients with craniopharyngiomas involving the hypothalamus, resting-state connectivity was disrupted not only in the HT but also in the CC and THA, indicating coordinated functional alterations across these structures.^103^ Congenital disorders such as septo-optic dysplasia further illustrate these midline associations, with concurrent abnormalities of the HT, OC, CC, and VT midline structures frequently observed.^104^ Neurodevelopmental conditions such as autism spectrum disorder show morphological alterations involving the OC, CC, and VT, which correlate with symptom severity and sensory behavior, suggesting abnormal remodeling of midline connectivity.^105^

Nevertheless, in the three-module framework, our work offers further evidence from the perspective of protein expression. This analysis revealed a conserved cortical integration module of 26 proteins that converge on synaptic signaling, vesicle trafficking, and cytoskeletal regulation—processes central to circuit integration and information processing in cortical tissue. The preservation of this module across all four cortical lobes suggests that it underlies common aspects of cortical specialization and increased cognitive function, whereas its perturbation may increase vulnerability to neurodevelopmental, neuropsychiatric, and seizure-related disorders. The cortical integration module is extensively interconnected, allowing for the integration and exchange of information through large-scale neural networks. This cross-regional information processing may depend on efficient synaptic transmission and neurotransmitter regulation. The observed upregulation of synapse-related processes in these regions likely supports the rapid flow of information between lobes, possibly ensuring the overall coherence of brain function. Although previous studies have suggested a correlation between CC thickness and intelligence, as well as its association with social behavior in mice,^48,106^ these findings have not been explored at the molecular level. While the CB, traditionally linked to motor control, has been implicated in cognitive and language functions—potentially owing to structural changes during human evolution—this expansion remains insufficiently supported by research.^107^ The evidence supporting the involvement of the BS and VT in higher-order cognitive functions is even more limited.

To address the existing gap in research on extracortical regions, our findings suggest that the midline regulatory axis and evolutionarily conserved regions (BS and CB) have been underappreciated in terms of their functional complexity. This proteomic map reveals their multifaceted roles in neurodevelopment, signaling integration, and cognitive regulation. The midline regulatory axis shows elevated activity in gliogenesis, synaptic assembly, energy metabolism, and embryonic development. At the subcellular level, this axis appears to be characterized by coordinated protein targeting to axons and vesicles, glial interactions, and receptor-mediated endocytosis, which may support neuronal connectivity, CSF homeostasis, and structural integrity. Within this framework, region-specific proteins may play distinct molecular roles: ARHGEF11 in THA/HT is involved in cytoskeletal remodeling and morphological regulation; S1PR1 in the CC mediates neurodevelopmental signaling and axonal pathfinding; and POSTN in the VT supports neuroprotection and structural development of the ventricular system. Together, these molecular signatures emphasize the axis’s role in higher-order regulation and maintenance of brain architecture. Similarly, evolutionarily conserved regions, including the CB and BS, are enriched in adaptive signaling and homeostatic coordination, contrary to their long-held classification as lower-order motor structures. CBs exhibit elevated expression of LMAN2L and EIF2AK2, proteins associated with intelligence, as well as MACROH2A2, an epigenetic regulator, suggesting functional involvement in cognition and gene‒environment interactions. In the BS, RASGRF2—a molecule critical for Ras-mediated signaling and synaptic plasticity—may implicate this region in memory regulation and neuromodulatory responses to environmental cues. These findings collectively indicate that CB and BS have far more complex functions than previously acknowledged.

When biological pathways, protein signatures, cellular components, and anatomical regions are integrated, we suggest that the midline regulatory axis may rely on several key elements to maintain its function. These include synaptic proteins that ensure proper neurotransmission, stable metabolic activity to supply energy, and ECM molecules that support structural integrity. Disruption of any of these components may contribute to the development of degenerative diseases or congenital, treatment-resistant disorders linked to this axis. These findings collectively suggest that proteomics offers a significant advantage in identifying cellular physiological processes, dynamic changes, and cross-level integrations, especially in studies related to structure and function. A combined proteomics and transcriptomics approach may provide a more comprehensive understanding of biological processes, compensating for individual limitations and enabling a deeper exploration of molecular mechanisms. By revealing complementary aspects of cellular physiology, proteomics focuses on the activity of functional proteins, whereas transcriptomics highlights gene expression regulation. Together, they provide a full picture of how cells respond to environmental changes at the molecular level. Transcriptomics may reveal gene expression changes that are not mirrored at the protein level owing to factors such as post-translational modifications or translation efficiency. Therefore, choosing appropriate omics techniques and combining them effectively will enable researchers to gain deeper insights into specific biological questions.

Our three-module framework reveals brain organization beyond anatomy. This approach strongly supports emerging research on AD, where multiple midline and deep brain structures—including those grouped within our axis—show correlated pathological changes, underscoring their functional and structural interdependence. For example, studies have highlighted early structural changes in the HT in AD patients, which may reflect secondary atrophy following volume loss in the HIP.^108^ These findings suggest that protein-level coordination among these regions may underlie their shared vulnerability to degeneration. Moreover, recent work on the choroid plexus has revealed significant inflammatory and metabolic alterations in AD, accompanied by measurable volume changes that correlate with glymphatic dysfunction, brain atrophy, tau pathology, synaptic loss, and cognitive decline.^109,110^ These findings position the choroid plexus—and by extension, the VT—as a central player in establishing a pathological milieu that impairs HIP and THA/HT function. Further multimodal neuroimaging studies have revealed linked functional and structural deficits across these regions in AD patients. Patients show reduced functional connectivity between the THA/HT and HIP, which is associated with decreased white matter integrity in the CC—a key commissural pathway.^111^ In parallel, increased mean diffusivity in the THA/HT and HIP indicates microstructural damage, reinforcing the notion that these regions are coaffected in the disease process.^111^ Visual pathway structures such as the OC and suprachiasmatic nucleus also participate in this coordinated pathology. While animal models show axonal degeneration in the optic tract without initial chiasmal involvement,^112^ the suprachiasmatic nucleus—the master circadian pacemaker—exhibits clear alterations in AD, contributing to sleep‒wake disturbances that exacerbate amyloid pathology and cognitive decline.^113^ Collectively, these AD-related findings validate the biological importance of our protein-based “midline regulatory axis”. They illustrate how functionally linked regions can exhibit coordinated changes in pathology, reinforcing the value of a molecular-level framework for disease.

On the basis of the novel proteomic signatures identified in this study, we propose a translational roadmap for clinical application. This framework aims to identify biomarkers for brain disorders with regional vulnerability. The differential protein expression across brain modules suggests diagnostic potential. For example, elevated POSTN in the VT may serve as a biomarker for congenital hydrocephalus or periventricular white matter injury. Similarly, S1PR1 overexpression in the CC can help stratify patients with neurodevelopmental or neuroinflammatory disorders such as callosal agenesis and multiple sclerosis. Proteins enriched in evolutionarily conserved regions—including LMAN2L and EIF2AK2 in the cerebellum and RASGRF2 in the brainstem—also represent promising fluid-based biomarkers for cognitive impairment and autism spectrum disorders. Several research directions are recommended to advance these findings. Initial work should validate key region-specific proteins such as POSTN, S1PR1, and CABP1 in well-characterized clinical cohorts. These cohorts should include retrospective samples and prospectively recruited patients with conditions such as congenital hydrocephalus or multiple sclerosis. Combining molecular data with neuroimaging and clinical phenotypes will strengthen biomarker‒disease associations. Subsequent studies should evaluate candidate detection methods in biofluids. Targeted mass spectrometry^114,115^ and ultrasensitive immunoassays^116^ can assess biomarker potential in cerebrospinal fluid and plasma. Further analysis should focus on evaluating the functional and druggable properties of candidate proteins. Computational screening against established druggability databases combined with network-based prioritization approaches could help identify high-priority targets.^117^ Experimental validation using disease models will then be essential to confirm their therapeutic relevance.

Our comparative analysis across the five brain regions common to both the HPA RNA dataset and our study—the cortex, BS, CB, AN, and HIP—revealed clear regional distinctions in protein‒RNA correlations. Although all regions present some degree of discordance in the transcriptome‒proteome, the underlying functional patterns differ markedly, underscoring the importance of region-specific interpretation.

In the cortex, proteomic data revealed strong enrichment of pathways involved in synaptic transmission, neuronal structure, energy metabolism, and higher-order cognitive functions, whereas only amine transport was enriched at the RNA level (Supplementary Fig. 10a). Similarly, in the BS, protein abundances related to energy metabolism, oxidative stress, and vascular development were notably elevated, whereas transcriptomic signals related to developmental and structural programs were more prominent (Supplementary Fig. 10b). The CB displayed a distinct profile, with proteomic enrichment in posttranscriptional and epigenetic regulation, whereas chromatin dynamics and electrophysiological modules were more visible at the RNA level (Supplementary Fig. 10c). In the HIP, neuronal development and immune pathways were highlighted in the proteome, in contrast to fluid and ion homeostasis, which were more prominent in the transcriptome (Supplementary Fig. 10e). A notable exception was observed in the AN, where synaptic transmission and higher-order neural functions—typically protein-enriched in other regions—were enriched primarily at the RNA level (Supplementary Fig. 10d). This finding suggests a unique regulatory strategy in this region, possibly reflecting its role in rapid emotional processing and dynamic transcriptional adaptation.

From these observations, a general principle emerges: the proteome appears to support immediate, functional demands—such as synaptic activity, energy production, and immune defense—through stable, preexisting protein pools that enable swift physiological responses. In contrast, the transcriptome often reflects longer-term regulatory planning, including developmental and homeostatic programs that require ongoing gene expression adjustments. This conceptual framework is supported by external biological evidence. For example, studies of circadian biology have demonstrated that core clock genes such as CLOCK, BMAL1, PER, and CRY are modulated by external cues such as feeding‒fasting cycles, with their protein activities often fine-tuned posttranslationally rather than through transcriptional changes alone. Similarly, exercise physiology research has repeatedly documented poor correlations between mRNA and protein levels of metabolic regulators such as PPARα, PPARγ, and PGC-1α, underscoring the importance of posttranslational control in metabolic adaptation. In summary, our findings highlight that brain region specialization cannot be fully understood through transcriptomics alone. Proteomic data reveal critical regulatory mechanisms—operating through translational efficiency, protein turnover, and posttranslational modifications—that are essential for obtaining a holistic view of brain function in health and disease.

One limitation of our study is the inability to identify or differentiate alternative pathophysiological mechanisms contributing to brain injury, such as direct viral toxicity, endothelial inflammatory damage, angiotensin receptor dysregulation, or immune dysregulation (e.g., cytokine release syndrome). As such, the molecular correlates of disease phenotypes across multiple brain regions remain unknown. Understanding how disease states alter protein expression will be critical to uncovering the underlying mechanisms of these symptoms and their associated brain functions.

Additionally, recent comparative transcriptomic analyses of living and postmortem human brains demonstrated that approximately 80% of prefrontal cortex genes exhibit expression differences between the two conditions.^118^ These discrepancies were not attributable to variations in RNA quality, cellular composition, postmortem interval, or technical factors and were consistently replicated across cohorts. This evidence suggests that postmortem transcriptional profiles may not fully reflect the molecular state of the living brain. However, previous studies have indicated that the proteome is more stable than RNA in the postmortem period. This evidence suggests that protein profiles may provide a more robust reflection of antemortem biological states than the more rapidly degrading transcriptome.^119–121^ Future work should integrate data from antemortem samples, rigorously controlled postmortem interval studies, and orthogonally validated assays.

A principal consideration is the use of FFPE-derived samples. Formalin fixation can cause protein cross-linking and chemical modifications. However, it is well documented that this effect has a relatively minor impact on proteomic studies.^122^ Reassuringly, the literature validates the use of FFPE tissues for proteomics, as there is a remarkable 89.9% overlap in protein identification between matched fresh-frozen and FFPE human brain tissues.^16^ Despite this high concordance, the limitation that formalin fixation might subtly alter the quantitative accuracy for a minority of proteins or obscure certain epitopes remains.

Furthermore, in interpreting the functional implications of proteomic findings, inferences based primarily on GO enrichment analyses and the expression patterns of individual proteins have inherent limitations. While these computational approaches provide valuable initial insights into potential biological processes, they remain susceptible to reverse inference and do not fully capture the complexity of neurobiological mechanisms. Future studies incorporating orthogonal approaches—such as spatial proteomics, cell type-specific validation, or functional imaging—will be essential to further corroborate and refine the proposed functional associations.

In our study, the comparison between the proteomic and transcriptomic datasets revealed only a limited correlation (Fig. 6b), which is consistent with findings reported in previous studies. Studies have consistently reported only modest correlations between the transcriptome and proteome levels, typically with r values of 0.4–0.7 across genes and 0.1–0.5 across samples. This mismatch is attributable to multiple layers of regulation. Evolutionarily, strict control of protein abundance is energetically costly; thus, proteins are generally more tightly regulated than are RNAs, and within proteins, those critical to essential functions are subject to stricter regulation.^123^ Although posttranslational regulation is wasteful, it allows rapid responsiveness and is therefore preferentially used in vital processes.^124^ Biologically, several mechanisms contribute to this decoupling. RNA interference by miRNAs and other noncoding RNAs can suppress translation, promote mRNA degradation, or increase protein synthesis. Protein levels are also shaped by turnover via the ubiquitin–proteasome and autophagy pathways.^125^

In addition, we conducted additional immunohistochemical (IHC) experiments to demonstrate that genes (such as SF3B4 and FMN2) that were not observed in the transcriptome (Supplementary Fig. 9b) are transcribed into proteins in brain tissues (Supplementary Fig. 8b). IHC experiments confirmed that the proteins validated by proteomics but not captured by transcriptomics are indeed present in brain tissues, thus representing the actual discrepancy between the proteomic and transcriptomic landscapes.

A potential concern raised regarding our study is that some conclusions related to brain regional specialization might be confounded by differences in cell-type composition rather than reflecting intrinsic regional properties. To address this, we performed IHC analysis on key candidate proteins (Supplementary Fig. 8), which confirmed that SF3B4, FMN2, and FMNL1 are specifically enriched in neuronal populations. These results provide direct evidence that our findings primarily reflect genuine biological specialization within principal neuronal cells, effectively alleviating the core concern of cell mixture artifacts. Nevertheless, we acknowledge that our current IHC-based validation, while robust, focuses on selected key proteins. Future studies could benefit from more definitive and high-resolution approaches, such as cell sorting combined with proteomic profiling or single-cell proteomics, to further refine the cell-type specificity of protein expression across brain regions.

This study provides a detailed regional proteomic map of the human brain, highlighting region-specific protein expression patterns and their functional roles. By analyzing 13 different brain regions, we identified distinct proteomic signatures for each, revealing the functional diversity across the brain. A three-module framework was proposed. While cortical regions align with established cognitive functions, the midline regulatory axis (thalamus/hypothalamus, corpus callosum, ventricles, optic chiasm) plays underexplored roles in neurodevelopment, interregional signaling, and structural homeostasis. These functions may be fundamentally dependent on efficient synaptic communication, robust energy metabolism, and the integrity of the extracellular matrix. Additionally, the cerebellum and brainstem exhibit functional potential in cognition and adaptive regulation. These findings offer new insights into normal brain physiology and set the stage for future research into brain function and its alterations in disease.

## Materials and methods

### Human subjects

Brain samples were obtained from eight autopsies, and the clinical data are detailed in Supplementary Tables 1 and 2. The autopsies were conducted in Wuhan, China, between February 18 and April 4, 2020. Each whole brain was excised at 8 hours postmortem and immediately fixed, ensuring uniform postmortem handling and consistent protein preservation across all cases. Therefore, all proteomic analyses were based on tissues uniformly fixed at the same postmortem time point, minimizing the potential influence of the postmortem interval on protein degradation or modification. The subsequent regional dissection of each fixed brain was performed 8–24 hours after fixation, while the tissue remained immersed in fixative solution.

All the subjects died from natural causes and were rigorously screened to exclude any conditions that might compromise brain homeostasis. The exclusion criteria were as follows: (1) documented neurological disorders (AD, Parkinson’s disease, cerebral ischemic stroke, epilepsy, demyelinating diseases, Huntington’s disease, amyotrophic lateral sclerosis, or multiple system atrophy); (2) major systemic diseases (hypertension, myocardial infarction, atherosclerosis, pneumonia, diabetes mellitus, hepatic/renal insufficiency, or autoimmune diseases); (3) neuropsychiatric conditions (e.g., major depression, bipolar disorder, or schizophrenia); (4) intracranial pathologies (brain tumors, traumatic brain injury, or neuroinfections); and (5) history of substance abuse (alcohol or nicotine). The absence of these conditions was verified through a comprehensive medical record review.

Brain samples were obtained from the Biobank of Southwest Hospital, Third Military Medical University (TMMU), with written informed consent.^126^ This study complied with the regulations of the National Health Commission of China and the ethical principles of the Helsinki Declaration.

### Sample preparation

#### Deparaffinization and dehydration

The formalin-fixed, paraffin-embedded (FFPE) tissues were placed into Eppendorf tubes and incubated in a 60 °C-metal bath for 10 minutes; then, they were soaked in 1 mL of xylene, vibrated for 10 minutes, and centrifuged at 14,000 rpm for 5 minutes. Finally, the supernatant was discarded. The entire xylene wash cycle was repeated a second time. The precipitate was successively immersed in 1 mL of 99% ethanol, 1 mL of 96% ethanol, and 1 mL of 70% ethanol; the mixture was shaken for 5 minutes and centrifuged at 14,000 rpm for 5 minutes. Finally, the supernatant was discarded. The entire ethanol wash cycle was repeated a total of 6 times.

#### Rehydration and lysis

The samples were soaked in distilled water, shaken for 10 minutes, and centrifuged at 14,000 rpm for 5 minutes; finally, the supernatant was discarded. This step should be performed carefully. The texture of the treated sample is relatively loose at this time, and it is not easy to stick to the test tube wall. Next, 100 µL of LB (50% trifluoroethanol (TFE), 300 mm Tris HCl) was added to each sample, which was then ultrasonicated (1 s on, 1 s off, 20 s). To ensure that the sample fully fused with the LB after ultrasonication, instantaneous centrifugation was used. The test tube was subsequently incubated in a 90 °C-metal bath for 90 minutes. After heating, the samples were carefully removed from the test tube, but not before the temperature of the metal bath dropped below 60 °C. Ultrasound was repeated until no solid was observed.

#### Reduction, alkylation, and digestion

The sample volume was replenished to 100 µL with mass spectrometry (MS)-grade water; 30 µL of the sample was measured, and a final concentration of 5 mM Dithiothreitol was added. The mixture was incubated for 20 minutes at room temperature and then alkylated in 25 mM iodoacetamide at room temperature for 20 minutes in darkness. For proteolysis, first, 70 µL of 10% TFE and 1 µg of trypsin were added, and the mixture was incubated at 37 °C for 16 h. Second, 1 µg of trypsin was added, and the mixture was incubated at 37 °C for 4 h. Enzymatic hydrolysis was terminated by the addition of 50 µL of 1% trifluoroacetic acid (TFA).

#### Peptide cleanup

The mixture was centrifuged at 14,000 rpm for 10 minutes; then, the supernatants were transferred to a clean tube, desalted through C18 cartridges (Beijing Qinglian Biotech Co., Ltd., Beijing, China), and finally vacuum-dried by SpeedVac.

### Peptide prefractionation by high-pH liquid chromatography (HPLC)

Pooled peptides were fractioned via high-performance liquid chromatography (HPLC) to reduce sample complexity. Briefly, peptides were dissolved in buffer A (2% acetonitrile (ACN), pH 9.5), loaded on an Xbridge C18 column (Waters, MA, USA; 4.6 mm ×100 mm; 130 Å particle size, 5 μm pore size) and eluted with a 70-minute gradient from 0 to 95% buffer B (98% ACN, pH 9.5) at a flow rate of 0.7 mL/min. Aliquots were combined into 24 fractions before MS analysis.

### Mass spectrometry

The samples were measured via an integrated high-performance EASY-nLC 1200 system (Thermo Fisher Scientific) coupled to a Q Exactive HF-X Orbitrap mass spectrometer (Thermo Fisher Scientific) via a nanoelectrospray ion source (Thermo Fisher Scientific). The purified peptides were redissolved in mobile phase A (20% ACN and 0.1% formic acid) and directly loaded onto a C18 nanocapillary analytical column (Beijing Qinglian Biotech Co., Ltd., Beijing, China; 150 µm × 150 mm, 100 Å particle size, 1.9 µm pore size). For the proteome profiling samples, peptides were separated on an analytical column over a 90-minute gradient (buffer A: 0.1% formic acid and 80% H_2_O; buffer B: 0.1% formic acid and 20% ACN) at a constant flow rate of 0.6 μL/min (0 to 15 minutes, 8% to 12% buffer B; 15 to 65 minutes, 12% to 30% buffer B; 65 to 80 minutes, 30% to 40% buffer B; and 81 to 90 minutes, 95% buffer B).

To acquire MS data, the data-independent acquisition (DIA) scan mode was used for single-shot samples, whereas the fractionated samples of the pool were acquired in the top 40 data-dependent acquisition (DDA) scan mode. Both acquisition schemes were combined with the same liquid chromatography gradient. The mass spectrometer was operated with Xcalibur software (Thermo Fisher). The DDA scan settings at the full MS level included an ion target value of 3 × 10^6^ charges in the 350–1500 m/z (mass‒charge ratio) range, with a maximum injection time of 80 ms and a resolution of 120,000 at m/z 200. At the MS/MS level, the target value was 5 × 10^4^ charges with a maximum injection time of 45 ms and a resolution of 15,000 at m/z 200. For MS/MS events only, precursor ions with 2–7 charges that were not on the 16 s dynamic exclusion lists were isolated in a 1.6 m/z window. Fragmentation was performed by higher-energy C-trap dissociation with a normalized collision energy of 27 eV. DIA was performed with one full MS event followed by 42 MS/MS windows in one cycle. The full MS settings included an ion target value of 3 × 10^6^ charges in the 350–1500 m/z range with a maximum injection time of 50 ms and a resolution of 60,000 at m/z 200. The DIA precursor windows ranged from 378 m/z (lower boundary of the first window) to 1345 m/z (upper boundary of the 42nd window). The MS/MS settings included an ion target value of 1 × 10^6^ charges for the precursor window with an Xcalibur-automated maximum injection time and a resolution of 30,000 at m/z 200.

### Proteomic MS/MS data processing

We used deep-learning augmented proteomics data analysis software (Spectronaut, version 14.9.201124.47784; Biognosys, Switzerland). The MS data of the fractionated pool (DDA MS data, 24 fractions) and the single-shot samples (DIA MS data) were used to generate a DDA library and direct DIA library, respectively, which were computationally merged into a hybrid library. The raw DIA data were subsequently processed on Spectronaut using the default settings. All searches were performed against the human SP UniProt reference proteome of canonical and isoform sequences with 20,350 entries. Studies have used carbamidomethylation as a fixed modification, acetylation of the protein N-terminus, and oxidation of methionines as variable modifications. Default settings were used for other parameters. In brief, a trypsin/P proteolytic cleavage rule was used, permitting a maximum of two miscleavages and a peptide length of 7–52 amino acids. Protein intensities were normalized via the “Local Normalization” algorithm in Spectronaut in local regression mode; the retention time prediction type was set to dynamic iRT and the correction factor for window 1. The mass calibration was set to “local”, and the decoy generation was set to “Inverse”. Interference correction at the MS2 level was enabled, removing fragments for quantification on the basis of the presence of interfering signals but maintaining at least three fragments for quantification. The FDR was estimated with the mProphet approach and set to 1% at both the peptide and protein levels.

### Immunohistochemistry

Immunohistochemical staining was performed to validate the regional expression patterns of selected candidate proteins. Brain tissues from the frontal lobe (FL), temporal lobe (TL), parietal lobe (PL), occipital lobe (OL), cerebellum (CB), brainstem (BS), thalamus (TH), optic chiasm (OC), corpus callosum (CC), hippocampus (HIP), ventricles (VT), and olfactory bulb/olfactory tract (OB/OT) were processed for paraffin embedding and sectioned at a thickness of 4 μm. The sections were deparaffinized in xylene, rehydrated through graded ethanol, and subjected to heat-induced antigen retrieval in citrate buffer (pH 6.0) at 95 °C for 15 min. Endogenous peroxidase activity was quenched with 3% hydrogen peroxide for 10 min, followed by blocking with 5% bovine serum albumin for 30 min at room temperature.

For protein validation, FMNL1, a protein highly expressed in the four cortical lobes, was examined across all the brain regions listed above. In addition, SF3B4 and FMN2—identified as enriched in cortical regions in Supplementary Fig. 7b—were specifically assessed in the FL, TL, PL, and OL. Primary antibodies were applied overnight at 4 °C at the following dilutions: SF3B4 (Proteintech, 1:600 dilution), FMN2 (Proteintech, 1:800 dilution), and FMNL1 (Proteintech, 1:800 dilution). After washing, the sections were incubated with HRP-conjugated secondary antibodies for 1 h at room temperature and visualized via DAB. Hematoxylin was used for nuclear counterstaining. The slides were dehydrated, mounted, and imaged under a bright-field microscope at 20× magnification (scale bar: 50 μm). All the staining was independently repeated three times.

### Statistical and bioinformatics analysis

We conducted all the preprocessing, statistics and most of the visualizations via the statistical software R (version 4.1.0). The identified proteins were quantified, normalized to the total protein concentration, multiplied by 10^6^ (ppm), and log2 transformed. One-way ANOVA was subsequently used to determine whether there were any statistically significant differences in the normalized intensities among the 13 brain regions. The maximum mean of the normalized intensity region of proteins was extracted, and the average normalized intensity of proteins in the remaining regions was calculated. The fold changes of proteins for each brain region were then calculated in comparison to the average expression of samples of the remaining 12 brain regions. We filtered proteins for which the Benjamini‒Hochberg adjusted *p* value from one-way ANOVA, after the consensus correlation between random effects (donors) was estimated via the duplicate correlation function in the limma package, was less than 0.01. The proteins with the highest mean normalized intensity and at least 1.5 times the mean normalized intensity of the remaining regions were considered region-specific. One-way ANOVA and random effect (donor) correction were used to determine the proteins whose expression was significantly different between brain regions via the R package limma (version 3.46.0). Differences for which the Benjamini‒Hochberg adjusted *p* value was < 0.01 and the fold change was > 1.5 were considered statistically significant.

The brain mRNA expression dataset detected by RNA-seq and reported as the mean normalized transcripts per million (nTPM) for each of the brain regions was downloaded from the Human Protein Atlas (HPA) (https://www.proteinatlas.org/humanproteome/brain/human+brain). To detect brain region-specific expressed mRNAs, nTPMs <1 were set to NA, and nTPMs >1 were transformed to log2 (nTPM) space. The mRNAs with a maximum log2 (nTPM) and at least two times the mean log2 (nTPM) of the remaining regions were considered region specific.

Scatter plots, as well as heatmaps of significant proteins, were generated via the R packages ggplot2 (version 3.3.5) and ComplexHeatmap (version 2.8.0, distance: Pearson, linkage: complete). Partial least squares discriminant analysis (PLS-DA) of the proteins whose values in each sample were valid was performed via the R package mixOmics (version 6.26.0). The clusterProfiler package (version 4.1.0) was used to annotate proteins according to biological processes, cellular components, and molecular functions within the GO and KEGG pathway analyses.

## Availability of data and materials

The proteomics dataset generated in this study has been publicly released. It can be accessed from ProteomeXchange Consortium via the iProX^127^ partner repository, with the links https://www.iprox.cn/page/project.html?id=IPX0004550000 or https://proteomecentral.proteomexchange.org/cgi/GetDataset?ID=PXD034484.

## Supplementary information

Supplementary Materials

Supplementary Table 1

Supplementary Table 2

Supplementary Table 3

Supplementary Table 4
