# Supplementary material for: Region-resolved proteomic map of the human brain: functional interconnections and neurological implications
Source: Signal Transduct Target Ther. 2026 Feb 4;11:43. doi: 10.1038/s41392-025-02554-8 (PMC12868004; doi:10.1038/s41392-025-02554-8)
Supplement: Supplementary file 1 — Supplementary Materials [file 41392_2025_2554_MOESM1_ESM.docx]

Supplementary Materials for

Region-resolved proteomic map of the human brain: functional interconnections and neurological implications paste

Pei-Pei Zhang^#^, Man-Sheng Li^#^, Jia Zhou^#^, Chu-Hong Zhu, Rui Tang, Zhi-Cheng He, Xiao-Hong Yao, Yi-Fang Ping, Dong-Fang Xiang, Le-Yong Tan, Yu-Jie Wang, Shuai Wang, Si-Si Li, Jie Ma, Yun-Ping Zhu*, Xiu-Wu Bian*, Ling Leng*

^#^ These authors contribute equally to this work

Correspondence to: zhuyunping@ncpsb.org.cn (Yun-Ping Zhu), bianxiuwu@tmmu.edu.cn (Xiu-Wu Bian), lengling@pumch.cn (Ling Leng).

**This PDF file includes:**

Figures. S1 to S10

Captions for Supplementary Table 1 to 4

**Other Supplementary Materials for this manuscript include the following:**

Supplementary Table 1 to 4

Supplementary Figures





Fig. S1 Visualization of quality control (QC) metrics.

**(a)** The peptide count distribution of proteins identified from 99 brain samples. The median peptide count of all identified proteins was 8. **(b)** The missing value rates distribution of all identified proteins. The median missing value rate was 16%. **(c)** Protein expression level ranking across brain regions. The plot depicts log_2_(intensity) as a function of protein rank, with different colors representing distinct brain regions (PL, HIP, VT, CC, THA, AN, OL, FL, BS, TL, OC, CB, OB/OT). **(d)** Intensity distribution of log_2_(intensity) for proteins across different brain regions, with each color corresponding to a specific region. **(e)** Intensity dynamic range of proteins identified from 13 brain regions. **(f)** Coefficient of variation (CV) of normalized intensities across 13 brain regions. Box plots illustrate the distribution of CV values for each region.





Fig. S2 Expression distribution analysis, principal coordinate analysis (PCoA), and partial least squares discriminant analysis (PLS-DA) of the proteome profile identified from 13 brain regions based on mass spectrometry.

**(a)** Box plots are given for the expression level of proteins identified from 13 brain region samples. **(b),** **(c)** PCA and PCoA score plot showing the separation between brain region samples using the first two latent variables (LVs). **(d)** PLS-DA score plot showing the separation between brain region samples using the first two LVs.


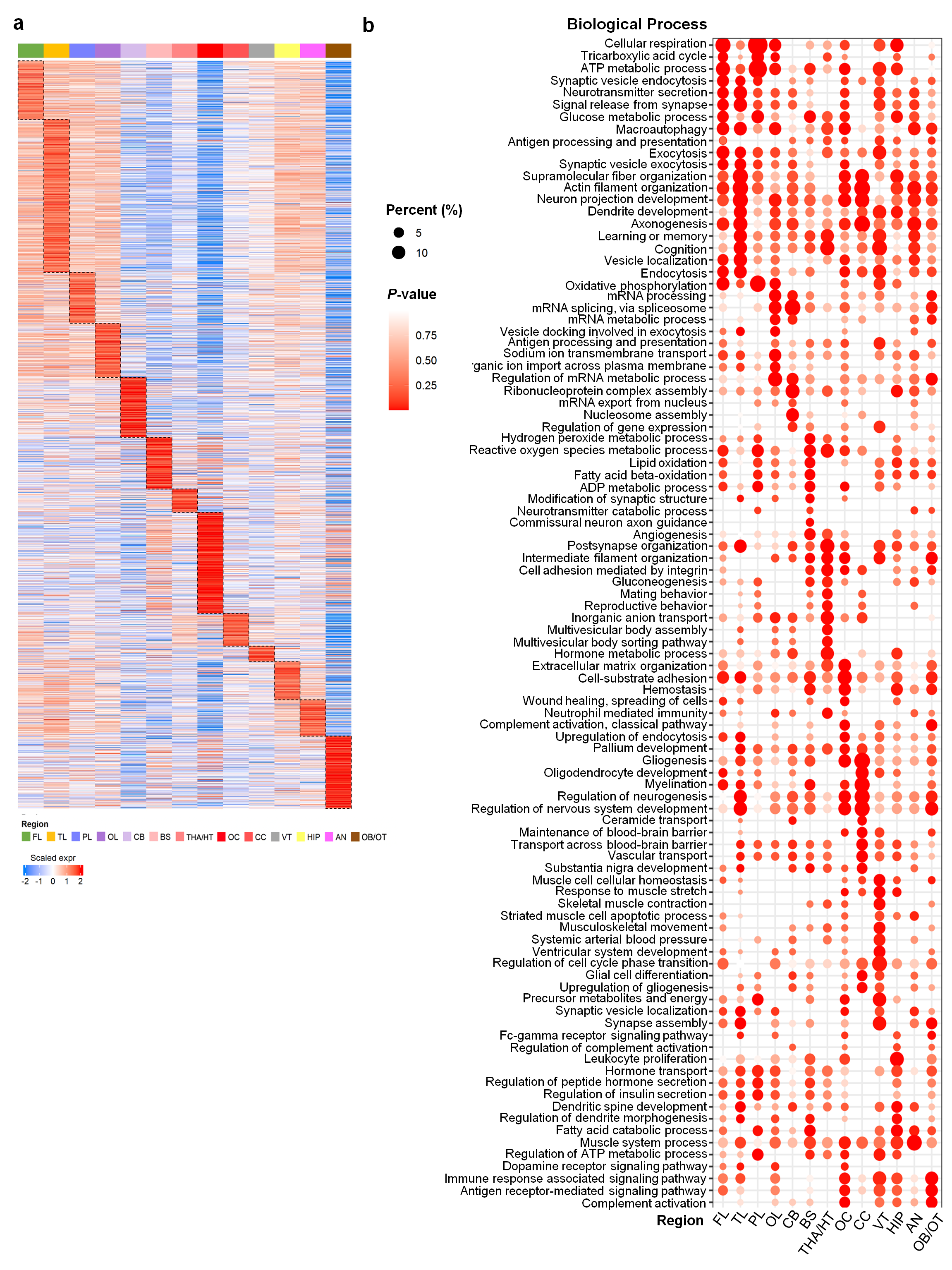


Fig. S3 Heatmap and cluster analysis of region-specific highly-expressed proteins.

**(a)** Heatmap of region-specific highly-expressed proteins for each brain region. Colors correspond to protein identity, with red and blue boxes indicating proteins with increased and decreased abundance, respectively. **(b)** Protein clusters and differentially expressed proteins allocated to 13 gene groups associated with similar biological processes in the 13 brain regions according to the degree of enrichment. Circles of different sizes represent the percentage of proteins included in each biological process. Boxes in gradient red indicate the degree of enrichment based on a *p*-value.


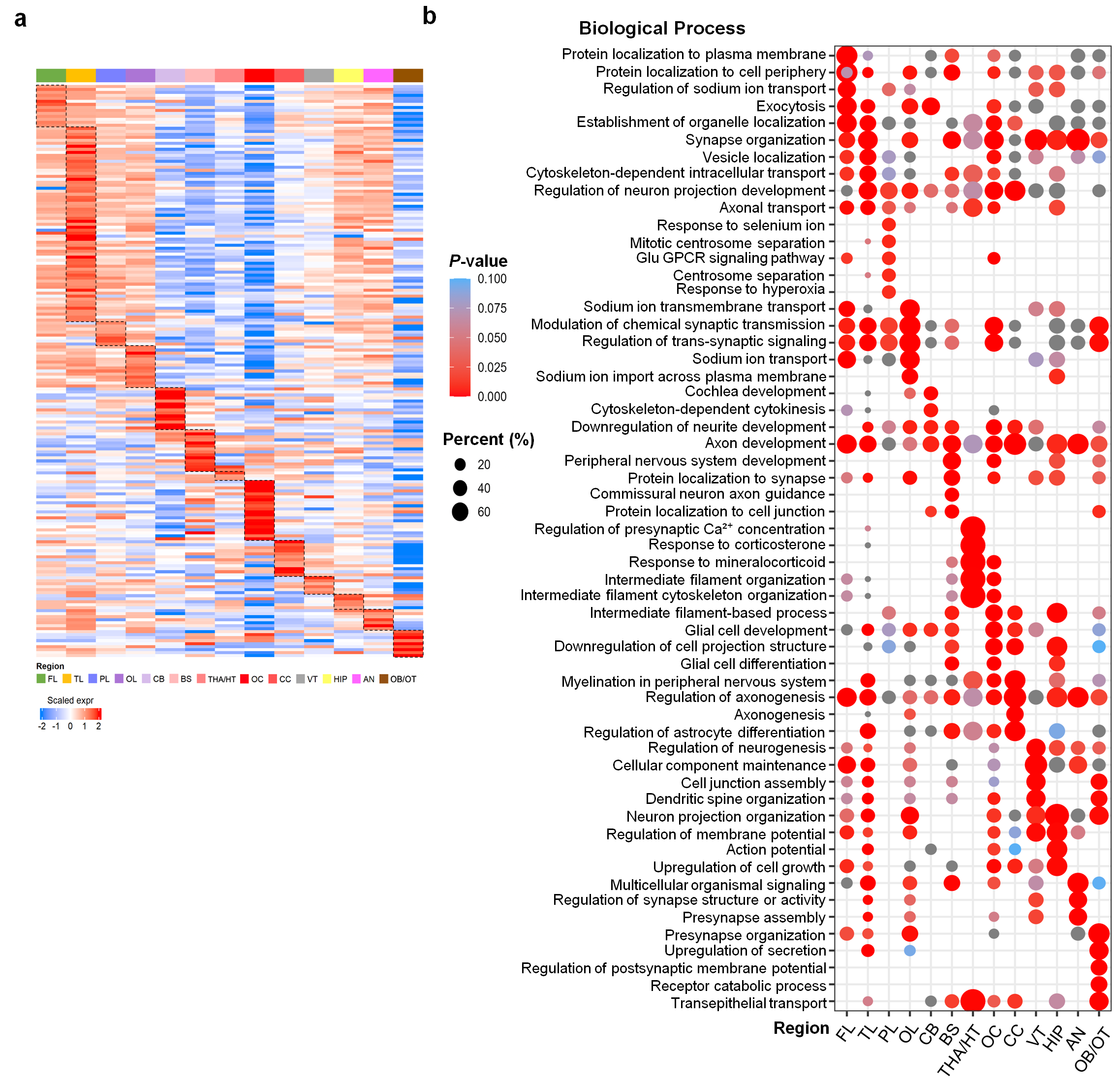


Fig. S4 Differentially expressed proteins in axons across brain regions.

**(a)** Heat map of the proteins differentially expressed in axons across 13 brain regions. Coexpression analysis of the protein modules obtained from 13 brain regions reveals their proteome specificity. The heat map displays z-scored normalized intensities of significantly differentially expressed proteins based on unsupervised hierarchical clustering. Colors correspond to protein identity, with red and blue boxes indicating proteins with increased and decreased abundance, respectively. **(b)** Cell signaling pathways involving region-specific highly-expressed proteins in axons across different brain regions. Circles of varying sizes represent the percentage of proteins included in each biological process. Boxes in gradient red indicate the degree of enrichment based on *p*-values.


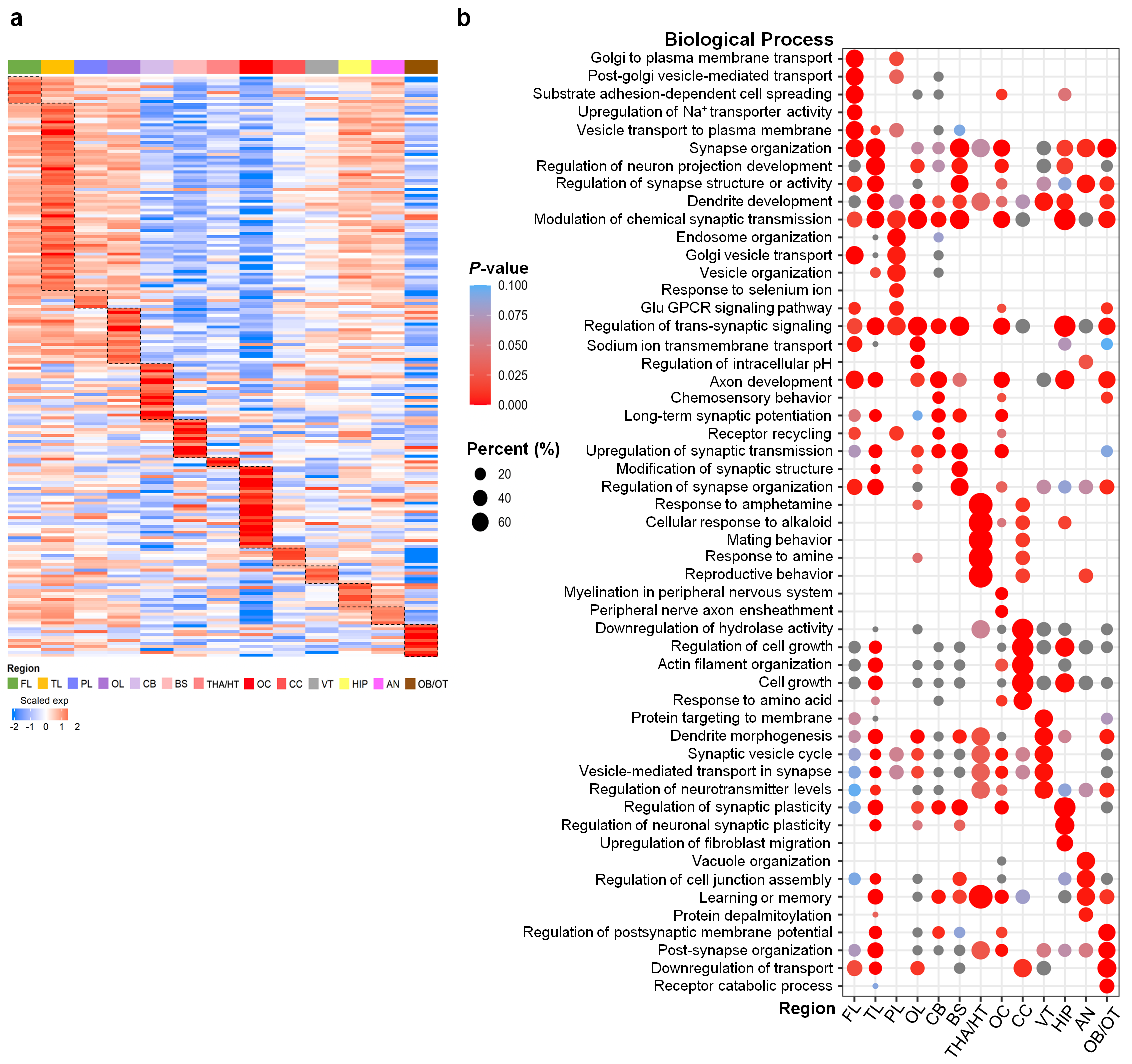


Fig. S5 Differentially expressed proteins in dendrites across brain regions.

**(a)** Heat map of the proteins differentially expressed in dendrites across 13 brain regions. A similar analysis as shown in Additional Figure S3 applies here. **(b)** Cell signaling pathways involving region-specific highly-expressed proteins in dendrites across different brain regions. Refer to Additional Figure S3 for details on the analysis methodology.


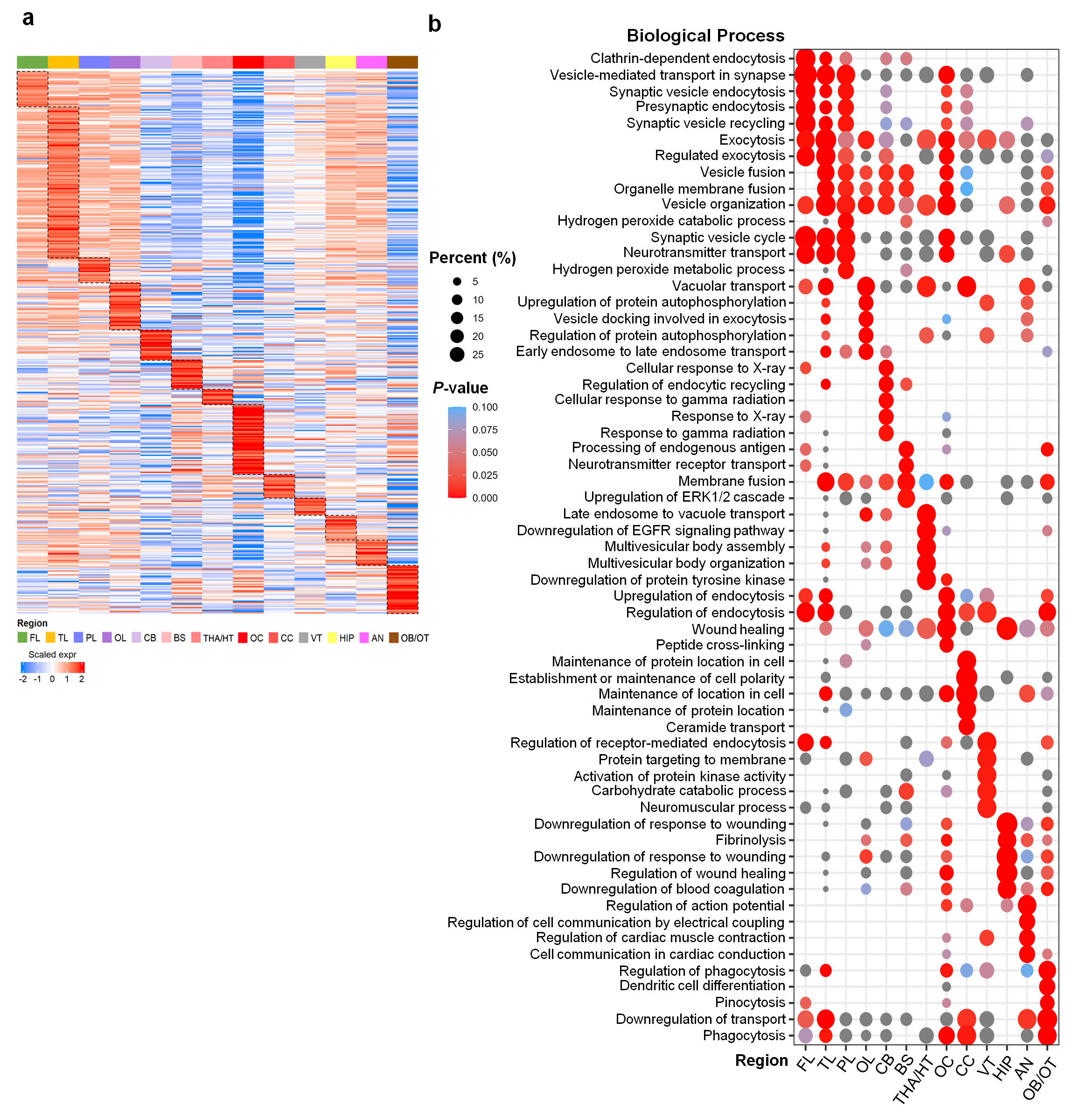


Fig. S6 Differentially expressed proteins in vesicles across brain regions.

**(a)** Heat map of the proteins differentially expressed in vesicles across 13 brain regions. A similar analysis, as shown in Additional Figure S3 applies here. **(b)** Cell signaling pathways involving region-specific highly-expressed proteins in vesicles across different brain regions. Refer to Additional Figure S3 for details on the analysis methodology.


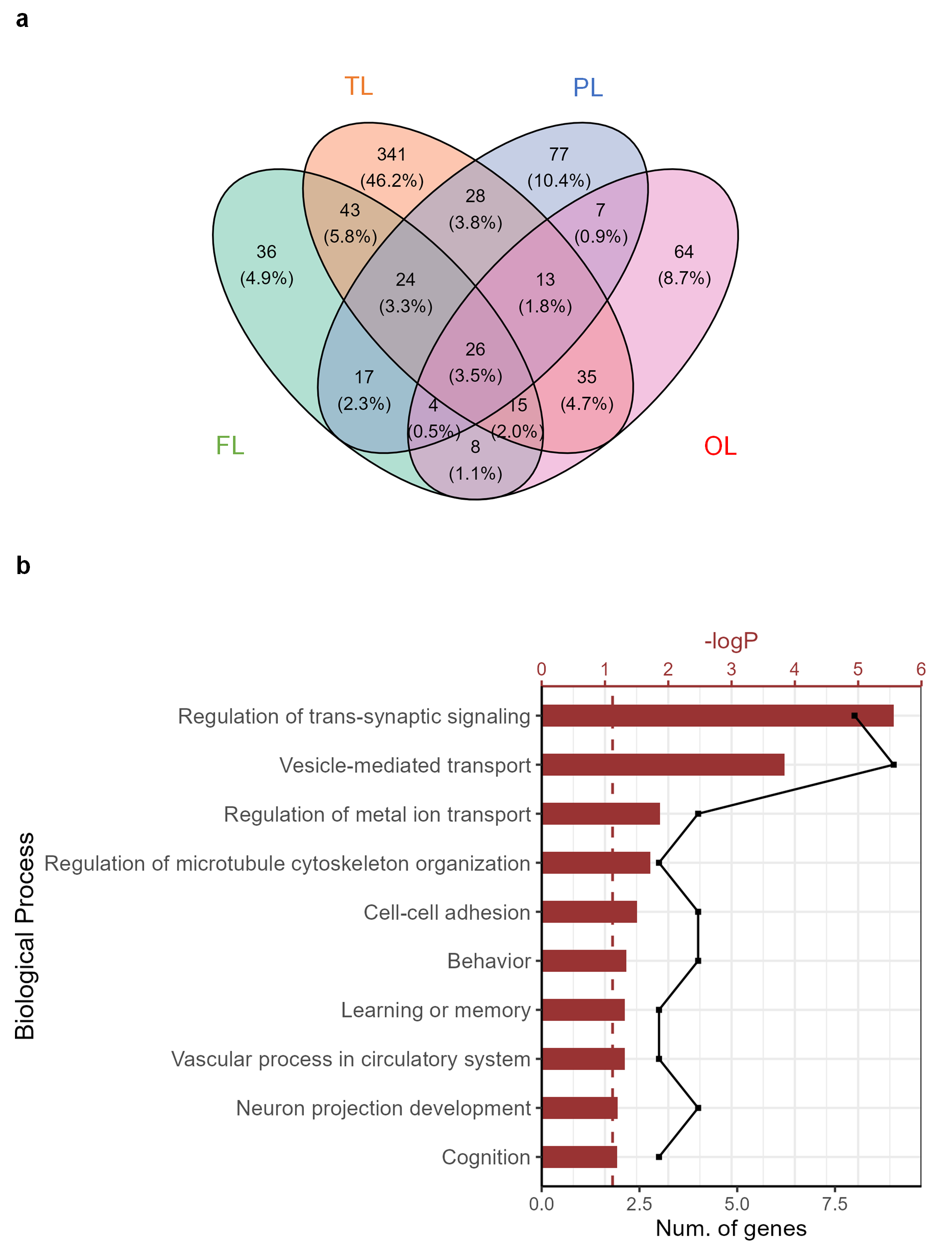


Fig. S7 Region-specific, highly-expressed proteins across four cerebral lobes compared to other non-cortical regions and their functional enrichment analysis.

**(a)** A Venn diagram illustrating the overlap of proteins specifically highly expressed in the frontal lobe (FL), parietal lobe (PL), temporal lobe (TL), and occipital lobe (OL) compared to other non-cortical brain regions. Notably, 26 proteins (3.5%) are commonly highly expressed across all four lobes. **(b)** A functional enrichment analysis of these 26 commonly highly-expressed proteins. The bar graph (upper y-axis) represents the statistical significance as -log_10_(*p*-value), and the line graph (lower y-axis) indicates the number of genes involved in each biological process.





Fig. S8 Immunohistochemical analysis confirming the region-specific expression patterns of key candidate proteins.

**(a)** FMNL1 showed low immunoreactivity in the cerebellum (CB), optic chiasm (OC), and olfactory bulb (OB), but higher staining intensity in other brain regions. Antibody: FMNL1 (Proteintech), dilution 1:800 (Scale bar: 50 μm). The experiment was repeated three times. **(b)** SF3B4 and FMN2 are positively expressed in the Frontal Lobe (FL), Temporal Lobe (TL), Parietal Lobe (PL), and Occipital Lobe (OL), and both are enriched in neuronal cells. Antibody: F3B4 (Proteintech), dilution 1:600; FMN2 (Proteintech), dilution 1:800 (Scale bar: 50 μm).


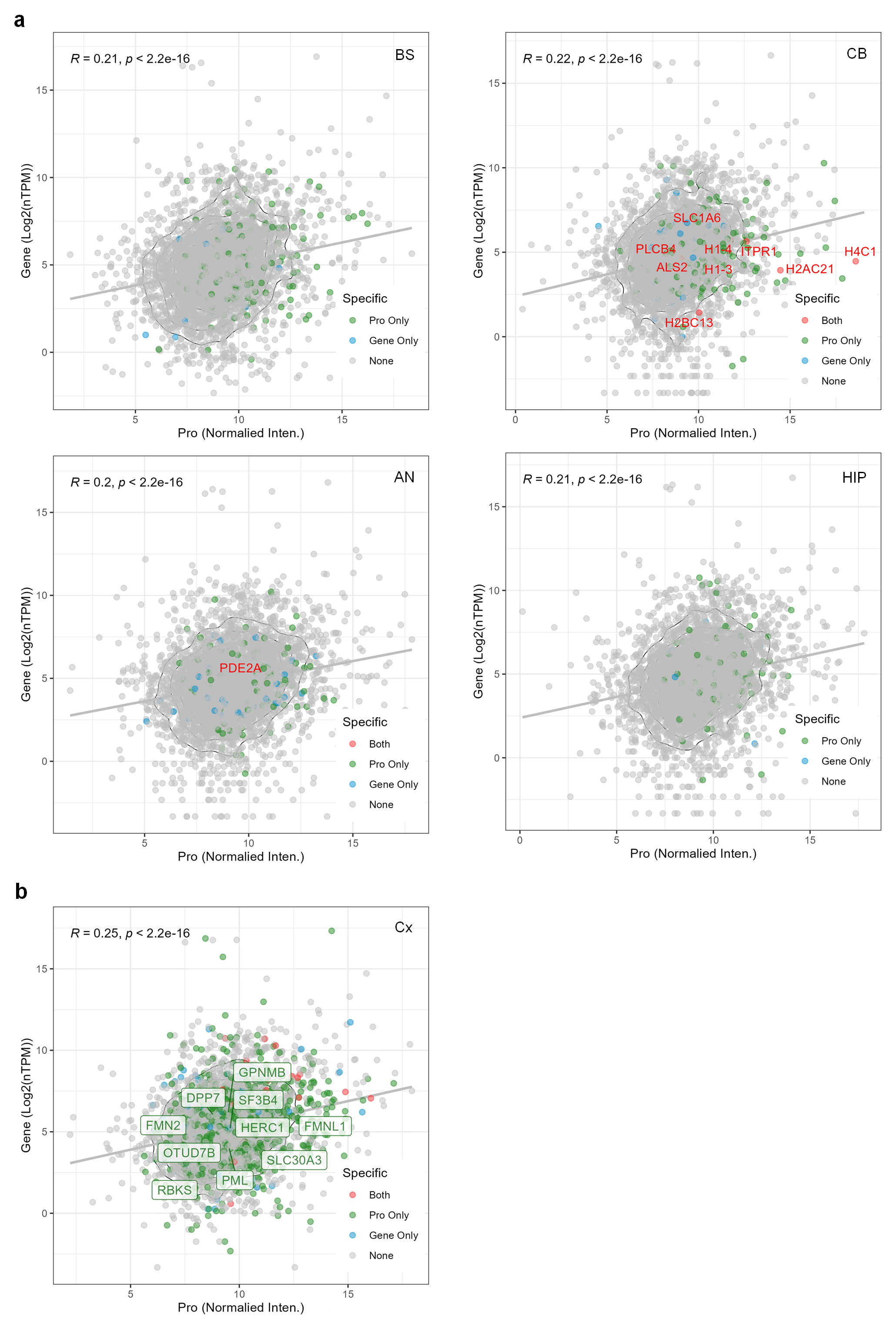


Fig. S9 The quantitative protein-RNA correlation per region.

**(a)** Genes marked in red are specific in both proteomics and transcriptomics. BS: Brainstem; CB: Cerebellum; AN: Amygdala; HIP: Hippocampus. **(b)** Genes marked in green are protein-specific only (unique to proteomics). Cx: Cortex. Scatter plots illustrate the Pearson correlation between protein normalized intensity (x-axis) and gene expression (log_2_(nTPM), y-axis) in five distinct brain regions. The correlation coefficient (*R*) and significance (*p*-value) are indicated for each region. Points are colored by specificity: red = *Both* (specific for both protein and gene), green = *Pro Only* (protein-specific), blue = *Gene Only* (gene-specific), and gray = *None* (non-specific).


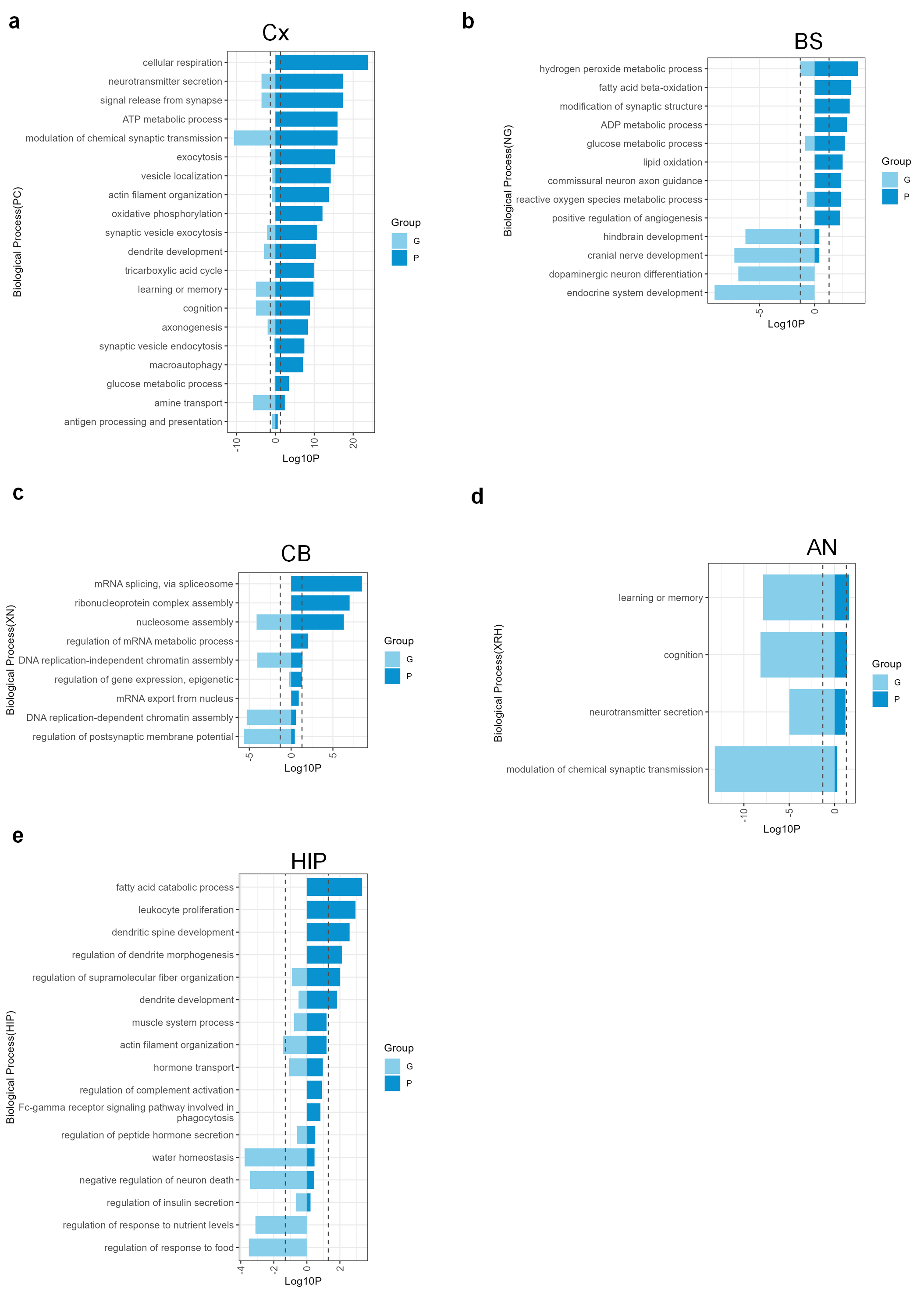


Fig. S10 Functional categories where proteomics adds unique signals.

Bar plots depict the enrichment of biological processes (y-axis) by −log_10_ (*p*-value, *x*-axis) in five brain regions, comparing gene (G) and proteomics (P) groups. This analysis highlights functional categories where proteomics contributes distinct signals, complementing transcriptomic insights. **(a)** Cx: Cortex. **(b)** BS: Brainstem. **(c)** CB: Cerebellum. **(d)** AN: Amygdala. **(e)** HIP: Hippocampus.

Supplementary Table 1. Overview of the characteristics of participants involved in the study.

Supplementary Table 2. The donors × regions sampling matrix.

Supplementary Table 3. All proteins identified in brain tissue samples from eight individuals based on 13 distinct regions of human brain.

Supplementary Table 4. Detailed list of region-specific, highly-expressed proteins identified in 13 brain regions.
